# Comorbidity in patients with cancer treated at The Christie

**DOI:** 10.1038/s41416-024-02838-w

**Published:** 2024-09-04

**Authors:** Azadeh Abravan, Corinne Faivre-Finn, Fabio Gomes, Marcel van Herk, Gareth Price

**Affiliations:** 1 Division of Cancer Sciences, The University of Manchester, Manchester, United Kingdom; 2 The Christie NHS Foundation Trust, Manchester, United Kingdom

**Keywords:** Oncology, Diseases

## Abstract

**Background:**

Comorbidities have been shown to impact the presentation and treatment of patients with cancers. This study investigates the prevalence and patterns of comorbidity in a pan-cancer cohort of patients treated at a large UK specialist cancer center over a 9-year period.

**Methods:**

A retrospective review of 77,149 patients from 01/01/2014 to 15/12/2022 was conducted using the Adult Comorbidity Evaluation 27 score (ACE-27) to assess the burden of comorbidities across 12 organ systems and an overall comorbidity burden. Binary and multinomial logistic regressions were utilized to evaluate the relationships between comorbidity incidence and demographic and socio-economic factors.

**Results:**

At the time of diagnosis, 59.7% of patients had at least one comorbidity, with the highest prevalence in lung cancer and the lowest in brain/CNS and endocrine gland cancers. Cardiovascular comorbidities were the most frequent. Comorbidity severity was higher in patients from more deprived areas. Age and performance status were associated with a higher incidence of all comorbidities examined. Patients with advanced stage had a lower risk of having a severe comorbidity burden.

**Conclusion:**

Comorbidities are common across all cancers but are more prevalent in certain patient populations. Further research to understand the implications of comorbidities in cancer management is needed.

## Background

Patients with cancer often present with a level of comorbidity at diagnosis, which can have a significant impact on their overall health, cancer care, and treatment outcomes [1]. Comorbidity, which encompasses psychological and physiological conditions, broadly refers to the presence of one or more health conditions that frequently coexist with a primary medical ailment [2]. It can impact the patient’s prognosis for a primary disease such as cancer. Comorbidity can have both positive and negative effects on the timing of cancer diagnosis [3]. Earlier diagnosis may happen when a comorbid patient is followed up routinely for their condition, while delayed diagnosis may occur when a patient or healthcare professional disregards cancer symptoms as they think it might come from pre-existing conditions [3–5]. Comorbidities can contribute to the progression of cancer through various mechanisms, such as inducing local and systemic inflammation and altering the tumor immune environment [3]. Comorbidities can affect the life expectancy and survival of the patients with cancer, independent of cancer stage and disease status [6, 7].

The prevalence of comorbidity in patients with cancer depends on both the cancer type and the studied population. For example, older cancer survivors are at high risk for multimorbidity [8], and as such patients diagnosed with lung or colorectal cancer tend to have a higher level of comorbidities, as these cancers often occur in older individuals. Some comorbidities are accompanied by an increased risk of developing certain types of cancer. For example, obesity and diabetes have been linked to an increased risk of developing colorectal, postmenopausal breast, and pancreatic cancer [9]. According to research conducted by Fowler et al. [10] and Balic et al. [11], diabetes, a prevalent comorbidity, has been associated with increasing the incidence rates of both liver and pancreatic cancer. On the other hand, as reported by Tsilidis et al. [12], diabetes was associated with reduced risk of prostate cancer.

Comorbidities can also pose challenges in the management of cancer. It can impact treatment decisions and the selection of appropriate treatment modalities. Following cancer diagnosis, patients with comorbidities are less likely to receive curative-intent treatments, thus compromising optimal care which can further lead to worse outcomes [13]. Furthermore, comorbidities restrict patients’ participation in clinical trials, especially those in early phases [14]. In summary, comorbidities can impact the effectiveness of cancer treatments, increase the risk of treatment-related complications, and contribute to overall mortality rates.

The existing body of evidence on this matter, however, is not consistently aligned. For instance, as well as the above noted disagreement on the impact of comorbidities on stage at diagnosis, some studies suggest that comorbidities do not lead to increased treatment toxicity [15, 16], implying that the presence of additional health conditions may not necessarily compromise the tolerability of cancer therapies. In contrast, other research findings indicate that comorbidities indeed contribute to heightened treatment toxicity [17], implying that these conditions may exacerbate the adverse effects of cancer treatments.

There are a number of methods to describe, measure, and quantify comorbidity such as the Kaplan-Feinstein, Charlson, Fleming, Keilhauer, and Klabunde comorbidity index and the Adult Comorbidity Evaluation 27 score (ACE-27) [18, 19]. The ACE-27 is the comorbidity tool developed through addition of important comorbid conditions to the Kaplan-Feinstein Comorbidity Index including dementia, diabetes mellitus, and acquired immunodeficiency syndrome (AIDS).

Adult Comorbidity Evaluation 27 (ACE-27): ACE-27 is a 27-item chart-based assessment method which evaluates the burden of comorbidities by reviewing 27 factors linked to 12 organ systems including cardiovascular, respiratory, gastrointestinal, renal, endocrine, neurologic, psychiatric, rheumatologic, immunologic, malignancy, substance abuse, and obesity. Specific conditions are scored into grade 1 (mild), grade 2 (moderate), or grade 3 (severe), according to the severity of individual organ decompensation and prognostic impact. The overall ACE-27 score is obtained based on the highest-ranking ailment(s) and can be none (0), mild (1), moderate (2) or severe (3). When two or more mild ailments are present, a final score of 1 (mild) is given. In situations where two or more moderate ailments are found in different organ systems or disease categories, the overall comorbidity score is classified as grade 3 (severe). During the ACE-27 assessment, the timeframe for reporting comorbidities varies by condition. For example, ventricular arrhythmias are classified as grade 3 if they occurred within the past 6 months and grade 2 if older. In contrast, conditions like respiratory issues and obesity depend on measurements such as forced expiratory volume in 1 s (FEV1) and body mass index (BMI), not time. More information on the classification can be found in ACE-27 UK values [20].

In general, there is an agreement that comorbidities are rather common among cancer patients [21]. However, this statement is dependent on many aspects such as patient’s physiological age and socio-economic status [22]. Other factors that can influence the presence and severity of comorbidity are the population, the measure of comorbidity utilized, and the cancer type [10]. It is evident that comorbidities play a crucial role in a patient’s cancer journey, and it is imperative to comprehensively consider their implications, given the significant uncertainty surrounding their exact impact on cancer care and outcome. While existing literature provides valuable insights, it primarily focuses on specific cancer sites and/or comorbidities, limiting our understanding of the broader picture. To our knowledge, our analysis is unique and the first of its kind, encompassing various cancer types and analyzing large scale real-world data from a cancer specialist center. The aims of this study were to 1) utilize an inclusive real-world data approach to assess the prevalence and severity of comorbidities among all patients with cancer treated over a 9-year period at a large specialist cancer center in the UK; 2) describe patterns of comorbidity with respect to patient characteristics including gender, age, and socio-economic status; and 3) investigate the inter-relationships of factors associated with differential comorbidity burden at the point of cancer diagnosis.

## Methods

### The Christie data

We queried data from patients diagnosed with cancer between 01/01/2010 and 15/12/2022 who were treated at The Christie NHS Foundation Trust in Manchester, UK. The initial query included available data on cancer sites (based on the International Classification of Diseases, 10th Revision (ICD-10) code), gender, age at diagnosis, and the Index of Multiple Deprivation. Next, we queried available data on Eastern Cooperative Oncology Group (ECOG) performance status, ACE-27 scores, and the date of the ACE-27 assessment, as the main focus of the study was comorbidity. It is important to note that ACE-27 and ECOG performance status, recorded in the ‘diagnosis and staging’ form completed during patients’ first consultations, are filled out by only a proportion of clinicians, leading to occasional missing entries. Additionally, 5% of patients choose to opt out of having their data used for research and audit purposes. In ACE-27 scoring, a category designated as 0 (none) signifies an absence of comorbidities. Here, missing entries are interpreted as ‘not available’ and are therefore excluded from the analyzed cohort, rather than implying the absence of comorbidities.

The patient’s comorbidity burden, assessed using the ACE-27 score, was documented during their initial consultation at The Christie, shortly after their referral. This assessment occurred before any anti-cancer treatment was administered at The Christie. Here, we concentrated only on ACE-27 score that existed prior to first treatment within our specified timeframe. In other words, in cases where individuals underwent multiple cancer treatments, we based the assessment of comorbidities on the first treatment episode. When patients had multiple recordings on the same date, we recorded the highest (i.e., worst) ACE-27 score and ECOG performance status.

The residential location at the time of diagnosis was utilized to infer patients’ socio-economic status, based on mapping of postcode to the combined indices of multiple deprivation score maintained by the UK Office for National Statistics [23]. The Index of Multiple Deprivation score was assigned at the Lower Layer Super Output Area level prior to being grouped into quintiles. The scores were derived across all domains included in the full index for the year 2019. This ranked score was split into quintiles to preserve privacy with the five-level, ordinal variable indicating the level of deprivation from the most deprived (Q1) to the least deprived (Q5).

Patient ethnic origin, stage, treatment intent, and type of treatment (systemic treatment, radiotherapy or a combination) were collected when available. The data on treatment included in this study include only treatments administered at The Christie and its associated satellite sites. Cancer sites are classified based on World Health Organization (WHO) guidelines using ICD-10 codes, as seen in Supplementary Table 1. It is important to highlight that in our dataset, missing data refers to information that was not collected or is unavailable for certain variables. Structured data were collected via an approved anonymous research database, the UK CAT Distributed Learning Database, with ethics approval granted by the UK North West-Haydock Research Ethics Committee (REC Reference: 21/NW/0347) and local consent obtained under Reference Number 2023-007. Censor date was set as 15/12/2022.

### Regional and national data

To compare the demographics and socio-economic factors of The Christie cohort with regional and national cancer data, the National Disease Registration Service (NDRS) was used, which records all cancer cases diagnosed in England and the corresponding Care Alliance in 2021 [24]. Data manipulation was conducted on the obtained data from NDRS to facilitate comparison with the studied cohort.

### Data analysis

We quantified the prevalence of overall scores and 12 comorbid conditions defined by ACE-27 as: 1) a crude measure, calculating the absolute counts and associated percentage of patients; and 2) an age-standardized measure [25], calculating the prevalence using the direct age-standardization of the cohort stratified by deprivation index quintiles and age range (using <40, 40–49, 50–59, 60–69, 70–79 and 80+ year-old age bands) to account for age differences in the population across different deprivation index quintiles.

An age-standardized measure is a statistical technique used to compare data across different populations by adjusting for variations in age distributions. We used it to allow for a fair and meaningful comparison of comorbidity prevalence between different socio-economic groups while accounting for inherent differences in the age structures of these sub-populations. For each age group and deprivation index quintile, the number of individuals who have the condition of interest (i.e. overall scores or any of the 12 comorbid conditions) was divided by the total population within that deprivation index quintile, regardless of the age distribution. In other words, we calculated age-specific rates in each age group and deprivation index quintile and used a standard age distribution (using the same cohort as a reference population) to weight these age-specific rates. We chose to use internal comparison with age-standardization within our population to account for unique characteristics of our study population, such as age distribution and deprivation quintiles, offering a more tailored analysis. This approach allowed us to minimize confounding factors that could differ between our population and external standards, ensuring a more accurate assessment of comorbidity prevalence in the cancer population analyzed. This approach, however, may limit the generalizability of our findings to broader populations.

Evaluation of the relationship between overall comorbidity burden (i.e., overall ACE-27 comorbidity score) and demographic including age groups, gender, cancer site, stage, and deprivation index, was conducted through multinomial logistic regression. Relationships between individual organ system comorbidity incidence and demographic and socio-economic factors were assessed using binary logistic regression.

Hierarchical clustering [26] using an agglomerative approach was employed to identify clusters of conditions in patients having more than one comorbidity. Correlation between the co-occurrence of different conditions was estimated using tetrachoric correlation. Tetrachoric correlation estimates the correlation between two binary variables assuming underlying continuous latent variables (i.e. the individual organ system comorbidity for a given ordinal comorbidity diagnosis). The degree of clustering was quantified using the agglomerative coefficient, with values closer to zero indicating well-formed clusters and values closer to one indicating less organized clusters.

Two-tailed *p*-values less than 0.05 were considered significant. Statistical analyses were performed in R 4.2.0 (R Core Team, Vienna, Austria).

## Results

Following the query on records of patients with available cancer ICD-10 codes, gender, age at diagnosis, and Index of Multiple Deprivation between 01/01/2010 and 15/12/2022 treated at The Christie NHS Foundation Trust, 123,568 records were identified. Of these, 97,469 records had available ACE-27 scores and ECOG performance status. After including only records from the earliest ACE-27 assessment date for patients with multiple records and selecting the highest (i.e., worst) ACE-27 score and ECOG performance status for patients with multiple recordings on the same date, 80,125 unique patients were identified. Details about the data selection process can be found in Supplementary Fig. 1.

We plotted the number of unique patients against the year of diagnosis, i.e., the year of ACE-27 assessment (Supplementary Fig. 2). As shown, the consistent collection of unique patient data in a structured format in our institute’s electronic patient record system began in 2014. Therefore, to minimize any bias in our analysis due to data availability, we excluded patients diagnosed between 01/01/2010 and 01/01/2014. A total of 77,149 unique patients diagnosed between 01/01/2014 and 15/12/2022 were included in the analysis (Supplementary Fig. 1). It is worth noting that there was a decline in cancer diagnoses in 2020, likely attributed to the COVID-19 pandemic (Supplementary Fig. 2).

To investigate any differences between patients with and without ACE-27, we limited the excluded data without ACE-27 to the period from 01/01/2014 to 15/12/2022, resulting in 22,099 unique patients (Supplementary Fig. 1).

**Fig. 1:**
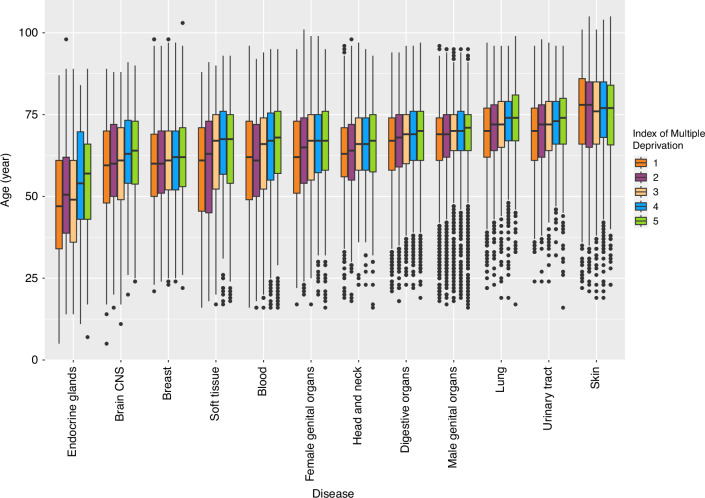
Box plots represent the distribution of age across different cancer sites, stratified by deprivation quintiles. Notably, individuals diagnosed with skin, lung, and urinary tract cancers tend to be older, whereas those with endocrine gland cancer are diagnosed at a younger age. The median age tends to increase when moving from the most deprived to the least deprived areas across all disease sites, with the exception of skin cancer.

**Fig. 2:**
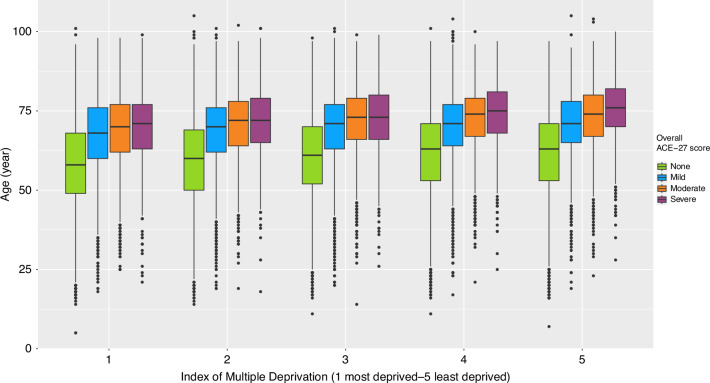
Box plots showing the distribution of age across overall ACE-27 scores, stratified by deprivation quintiles. Notably, the severity of ACE-27 scores demonstrates a consistent upward trend with advancing age across all deprivation quintiles. Additionally, when examining individual comorbidity scores, a discernible pattern emerges where age tends to incrementally rise from the most deprived areas to the least deprived areas. These findings underscore the complex interplay of age and socio-economic factors in shaping comorbidity burdens among the studied population. ACE-27 Adult Comorbidity Evaluation 27.

Across all patients the median age was 68 years (range 5–105 years) and 52.1% were female. In terms of ethnicity, the majority of patients (82.4%) for whom this information was available (66.4% of patients) identified as white British (54.7% of all patients). Additionally, 5.6% of all patients did not disclose their ethnicity. Demographics of the included patients are presented in Table 1. 29.4% and 17.9% of the patients live in the most and least deprived areas, respectively. At least one ACE-27 comorbidity was present in 59.7% of the patients. The most prevalent cancer undergoing treatment was breast (22.8%), followed by digestive organs (18.6%), male genital organs (16.1%), and lung cancer (14.9%). In general, patients with skin, lung, and urinary tract cancers were diagnosed at an older age with median (range) of 77 years (19–105), 72 years (17–99), and 72 years (16–98), respectively, while patients with endocrine glands cancer were diagnosed at a younger age (median 51 years, range 5–98 years) (Fig. 1). The median age generally rose as one transition from the most deprived regions to the least deprived areas across all cancer sites, except for cases of skin cancer (Fig. 1). Among all the patients with treatment intent available, 64.3% intended to receive curative-intent treatment and 30.9% scheduled to receive palliative-intent treatment. 4.2% of the patients were awaiting a treatment decision (Table 1). The severity of overall ACE-27 scores increases with age across all deprivation quintiles (Fig. 2). Moreover, for each comorbidity score, the age of individuals tends to rise from the most deprived areas to the least deprived areas (Fig. 2).

**Table 1:** Demographics of the included patients, presented for the total population and stratified by gender.

Factors	Levels	All (*n* = 77,149)	Female (*n* = 40,203)	Male (*n* = 369,46)
Age	Mean (SD)	65.9 (13.1)	64.1 (13.9)	67.9 (12.0)
Age group	<40	3005 (3.9)	1951 (4.9)	1054 (2.9)
	40–49	5488 (7.1)	4063 (10.1)	1425 (3.9)
	50–59	13,461 (17.4)	8296 (20.6)	5165 (14.0)
	60–69	21,295 (27.6)	10,439 (26.0)	10,856 (29.4)
	70–79	23,414 (30.3)	10,203 (25.4)	13,211 (35.8)
	80+	10,486 (13.6)	5251 (13.1)	5235 (14.2)
ECOG performance status	0	32,792 (42.5)	18,182 (45.2)	14,610 (39.5)
	1	25,691 (33.3)	12,748 (31.7)	12,943 (35.0)
	2	11,586 (15.0)	5670 (14.1)	5916 (16.0)
	3	6372 (8.3)	3216 (8.0)	3156 (8.5)
	4	708 (0.9)	387 (1.0)	321 (0.9)
Disease	Blood	1840 (2.4)	832 (2.1)	1008 (2.7)
	Brain CNS	1151 (1.5)	486 (1.2)	665 (1.8)
	Breast	17,591 (22.8)	17,496 (43.5)	95 (0.3)
	Digestive organs	14,352 (18.6)	5603 (13.9)	8749 (23.7)
	Endocrine glands	772 (1.0)	540 (1.3)	232 (0.6)
	Female genital organs	4813 (6.2)	4810 (12.0)	3 (0.0)
	Head and neck	4446 (5.8)	1440 (3.6)	3006 (8.1)
	Lung	11,524 (14.9)	5668 (14.1)	5856 (15.9)
	Male genital organs	12,409 (16.1)	3 (0.0)	12,406 (33.6)
	Skin	3816 (4.9)	1737 (4.3)	2079 (5.6)
	Soft tissue	1318 (1.7)	603 (1.5)	715 (1.9)
	Urinary tract	3117 (4.0)	985 (2.5)	2132 (5.8)
Deprivation index quintiles	1 (most deprived)	22,676 (29.4)	11,743 (29.2)	10,933 (29.6)
	2	14,367 (18.6)	7570 (18.8)	6797 (18.4)
	3	11,790 (15.3)	6193 (15.4)	5597 (15.1)
	4	14,537 (18.8)	7560 (18.8)	6977 (18.9)
	5 (least deprived)	13,779 (17.9)	7137 (17.8)	6642 (18.0)
Overall ACE-27 score	0 (none)	31,113 (40.3)	18,233 (45.4)	12,880 (34.9)
	1 (mild)	26,638 (34.5)	13,440 (33.4)	13,198 (35.7)
	2 (moderate)	13,383 (17.3)	5899 (14.7)	7484 (20.3)
	3 (severe)	6015 (7.8)	2631 (6.5)	3384 (9.2)
Cardiovascular system	Absent	47,776 (61.9)	27,401 (68.2)	20,375 (55.1)
	Present	29,373 (38.1)	12,802 (31.8)	16,571 (44.9)
Respiratory system	Absent	66,788 (86.6)	34,853 (86.7)	31,935 (86.4)
	Present	10361 (13.4)	5350 (13.3)	5011 (13.6)
Endocrine system	Absent	68,381 (88.6)	36,383 (90.5)	31,998 (86.6)
	Present	8768 (11.4)	3820 (9.5)	4948 (13.4)
Gastrointestinal system	Absent	75,192 (97.5)	39,329 (97.8)	35,863 (97.1)
	Present	1957 (2.5)	874 (2.2)	1083 (2.9)
Renal system	Absent	76,038 (98.6)	39,693 (98.7)	36,345 (98.4)
	Present	1111 (1.4)	510 (1.3)	601 (1.6)
Neurologic system	Absent	72,156 (93.5)	38,026 (94.6)	34,130 (92.4)
	Present	4993 (6.5)	2177 (5.4)	2816 (7.6)
Psychiatric system	Absent	74,678 (96.8)	38,634 (96.1)	36,044 (97.6)
	Present	2471 (3.2)	1569 (3.9)	902 (2.4)
Rheumatologic system	Absent	74,376 (96.4)	38,387 (95.5)	35,989 (97.4)
	Present	2773 (3.6)	1816 (4.5)	957 (2.6)
Immunologic system	Absent	77,016 (99.8)	40,178 (99.9)	36,838 (99.7)
	Present	133 (0.2)	25 (0.1)	108 (0.3)
Substance abuse	Absent	76,091 (98.6)	39,863 (99.2)	36,228 (98.1)
	Present	1058 (1.4)	340 (0.8)	718 (1.9)
Malignancy	Absent	71,054 (92.1)	37,010 (92.1)	34,044 (92.1)
	Present	6095 (7.9)	3193 (7.9)	2902 (7.9)
Obesity	Absent	76,001 (98.5)	39,393 (98.0)	36,608 (99.1)
	Present	1148 (1.5)	810 (2.0)	338 (0.9)
T stage	T0	141 (0.2)	93 (0.2)	48 (0.1)
	T1	14,413 (18.7)	9573 (23.8)	4840 (13.1)
	T2	16,002 (20.7)	8034 (20.0)	7968 (21.6)
	T3	11,424 (14.8)	4098 (10.2)	7326 (19.8)
	T4	6296 (8.2)	2782 (6.9)	3514 (9.5)
	TX	1055 (1.4)	462 (1.1)	593 (1.6)
	Tis	349 (0.5)	245 (0.6)	104 (0.3)
	missing	27,469 (35.6)	14,916 (37.1)	12,553 (34.0)
N stage	N0	33,817 (43.8)	17,377 (43.2)	16,440 (44.5)
	N1	9575 (12.4)	5767 (14.3)	3808 (10.3)
	N2	5824 (7.5)	2763 (6.9)	3061 (8.3)
	N3	2302 (3.0)	1237 (3.1)	1065 (2.9)
	missing	25,631 (33.2)	13,059 (32.5)	12,572 (34.0)
M stage	M0	51,747 (67.1)	28,037 (69.7)	23,710 (64.2)
	M1	13,459 (17.4)	5645 (14.0)	7814 (21.1)
	missing	11,943 (15.5)	6521 (16.2)	5422 (14.7)
Stage	local	27,735 (35.9)	13,697 (34.1)	14,038 (38.0)
	loco-regional	15,817 (20.5)	8864 (22.0)	6953 (18.8)
	distant	13,459 (17.4)	5645 (14.0)	7814 (21.1)
	missing	20,138 (26.1)	11,997 (29.8)	8141 (22.0)
Treatment intent	Curative	49,602 (64.3)	28,002 (69.7)	21,600 (58.5)
	Palliative	23,819 (30.9)	10,494 (26.1)	13,325 (36.1)
	Awaited	3221 (4.2)	1442 (3.6)	1779 (4.8)
	missing	507 (0.7)	265 (0.7)	242 (0.7)
Treatment regimen	Radiotherapy	37,699 (48.9)	20,099 (50.0)	17,600 (47.6)
	Systemic therapy	4712 (6.1)	2594 (6.5)	2118 (5.7)
	Systemic therapy+ radiotherapy	6872 (8.9)	4148 (10.3)	2724 (7.4)
	missing	27,866 (36.1)	13,362 (33.2)	14,504 (39.3)
Patient ethnic origin	A - White British	42,207 (54.7)	21,724 (54.0)	20,483 (55.4)
	B - White Irish	842 (1.1)	403 (1.0)	439 (1.2)
	C - Any other White background	1050 (1.4)	610 (1.5)	440 (1.2)
	D - Mixed White and Black Caribbean	202 (0.3)	107 (0.3)	95 (0.3)
	E - Mixed White and Black African	114 (0.1)	57 (0.1)	57 (0.2)
	F - Mixed White and Asian	81 (0.1)	50 (0.1)	31 (0.1)
	G - Any other mixed background	113 (0.1)	74 (0.2)	39 (0.1)
	H - Indian or British Indian	338 (0.4)	187 (0.5)	151 (0.4)
	J - Pakistani or British Pakistani	718 (0.9)	417 (1.0)	301 (0.8)
	K - Bangladeshi or British Bangladeshi	100 (0.1)	58 (0.1)	42 (0.1)
	L - Asian - other	201 (0.3)	117 (0.3)	84 (0.2)
	M - Black Caribbean or Black British Caribbean	236 (0.3)	110 (0.3)	126 (0.3)
	N - Black African or Black British African	181 (0.2)	83 (0.2)	98 (0.3)
	P - Any other Black background	45 (0.1)	24 (0.1)	21 (0.1)
	R - Chinese	171 (0.2)	98 (0.2)	73 (0.2)
	S - Any other ethnic group	368 (0.5)	229 (0.6)	139 (0.4)
	Z - Not Stated	4295 (5.6)	2421 (6.0)	1874 (5.1)
	Not Set	25,897 (33.6)	13,434 (33.4)	12,453 (33.7)

ECOG performance status: Eastern Cooperative Oncology Group performance status; ACE-27: Adult Comorbidity Evaluation 27. TNM mapping: Local: N0 and T0-3, Loco-regional: T4 and any N, N1-3 and any T, Distant: any M1.

Significant disparities in the overall burden of ACE-27 score were identified among cancer groups (Supplementary Fig. 3). For instance, the percentage of patients who presented with no comorbidity at the time of their first appointment was highest in brain/Central Nervous system (CNS) (61.7%) and endocrine glands (61.3%) cancers, while it was lowest in lung (21%) and urinary tract (30.9%) cancers. On the other hand, the percentage of patients who presented with a severe overall score was highest in lung (19.7%) and skin (12.4%) cancers, and lowest for endocrine glands (1.8%) and breast (2.6%) cancers.

**Fig. 3:**
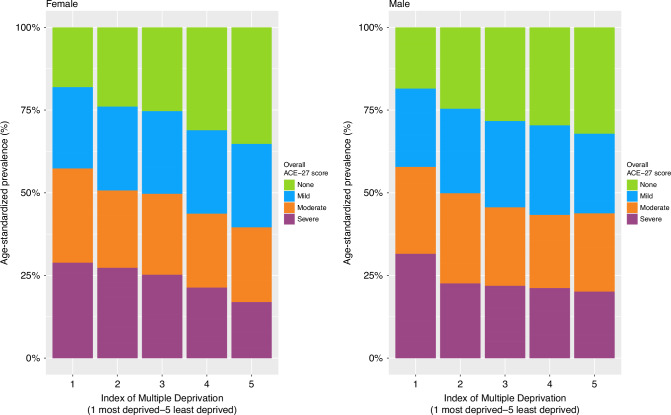
Age-standardized prevalence of overall ACE-27 scores, stratified by deprivation quintiles for females (left) and males (right). Of note, as individuals transition from the most deprived areas to the least deprived areas, a consistent reduction in comorbidity severity is evident. This trend holds true for both females and males. ACE-27 Adult Comorbidity Evaluation 27.

Across all patients, the age-standardized prevalence of a severe overall ACE-27 score was 31.3% in the most deprived areas and 17% in the least deprived areas (Supplementary Fig. 4). As one transitions from the most to the least deprived areas, there is a reduction in the severity of comorbidities in the full cohort and for both females and males (Supplementary Fig. 4 and Fig. 3).

**Fig. 4:**
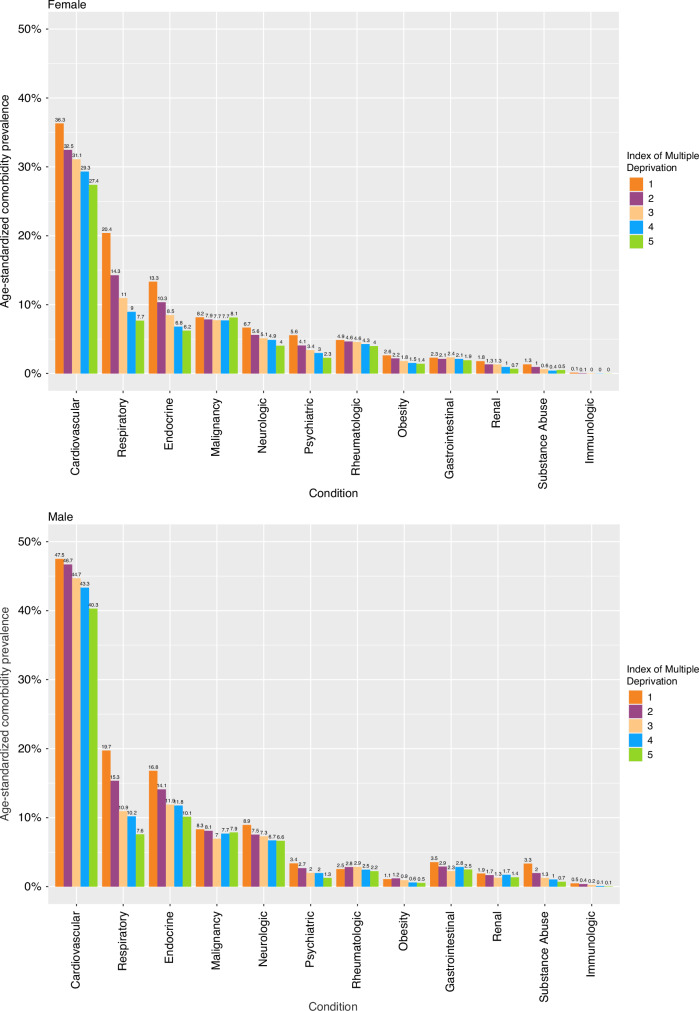
The age-standardized prevalence of 12 organ systems among male and female cancer patients, stratified by deprivation quintiles. These prevalences tend to be lower in less deprived areas (Q5) compared to the most deprived areas (Q1). Cardiovascular comorbidity was the most common in both genders. While males generally had higher prevalence rates, exceptions included psychiatric, rheumatologic, and obesity conditions. Previous malignancy prevalence was similar between genders. This analysis highlights gender-specific variations in condition prevalence, aiding our understanding of gender influences comorbidity distribution. Note that a patient can have more than one condition.

As presented in Fig. 4, cardiovascular comorbidity, including hypertension, emerges as the most common condition in both females and males. The age-standardized prevalence rates for the 12 organ systems were generally higher among males, with the exceptions of psychiatric, rheumatologic, and obesity conditions. The prevalence of respiratory condition and previous malignancy displayed a similar occurrence between females and males. The age-standardized prevalence of the 12 organ systems defined by ACE27 showed a tendency for the prevalence of these conditions to decrease in the least deprived areas (Q5) when compared to the most deprived areas (Q1) in both females and males (Fig. 4).

When investigating the age-standardized prevalence of the 12 organ systems for each cancer site, it was found again that cardiovascular comorbidity was the most common condition in both females and males (Supplementary Fig. 5). The distribution of the second most prevalent condition varied among different cancer sites and genders. Among head and neck and lung cancers in both females and males, respiratory disease presented as the second most prevalent condition. Also, in breast and soft tissue cancers among females, respiratory disease emerged as the second most prevalent condition. For other disease sites, endocrine conditions ranked as the second most prevalent condition in males. In females, the second most prevalent condition was either endocrine or previous malignancy, depending on the specific cancer site.

The age-standardized prevalence for individual cardiac conditions across all cancer sites are presented in Supplementary Fig. 6. The prevalence of cardiac conditions varied across cancer sites and was generally higher in males compared to females. Hypertension was by far the most prevalent cardiac condition in both females and males across all cancer sites. Additionally, the second and third most prevalent cardiac conditions were either arrhythmia or angina/coronary artery disease, with the exception of head and neck cancers in males and endocrine cancers in females, where myocardial infarction emerged as the second most prevalent condition.

Multivariable binary logistic regressions showing the associations between the ACE-27 organs systems (with incidence of 5% or higher) and age group, gender, ECOG performance status, stage, and deprivation index quintiles, are presented in the Supplementary Tables 2–6. Age group and ECOG performance status were found to have a positive association with the incidence of comorbidities in all five organ systems examined. Being male was associated with an increased risk for the incidence of all organ systems tested, except for respiratory and previous malignancy. Additionally, the deprivation index quintiles showed a significant correlation with the incidence of cardiovascular, respiratory, and endocrine systems. The least deprived quintiles were associated with the higher chance of having a previous malignancy. Except for endocrine condition, there was a lower risk of having the condition for patients diagnosed with loco-regional or metastatic disease compared with local disease.

**Fig. 5:**
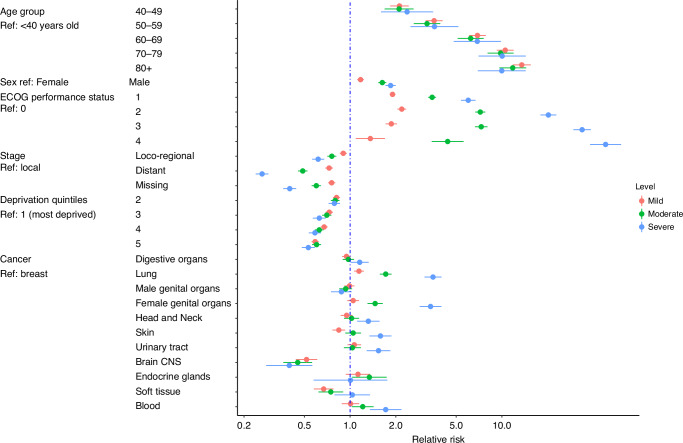
Forest plot representing the associations of overall ACE-27 comorbidity burden (reference: none) with factors including age group, gender, performance status, stage, deprivation index quintiles, and cancer site. These associations are derived from multivariable multinomial logistic regression models for the total cohort. ECOG performance status Eastern Cooperative Oncology Group performance status, ACE-27 Adult Comorbidity Evaluation 27.

The association between overall comorbidity burden in all cancer patients and factors including age groups, gender, performance status, disease site, stage, and deprivation index quintiles was assessed using multivariable multinomial logistic regression and is presented in the forest plot in Fig. 5. Of note, the risk of having a level of comorbidity increased with age. The odds of having higher levels of comorbidity (compared with none) increased for male compared with female. The odds of having higher levels of comorbidity increased with worse ECOG performance status, with higher odds of severe comorbidity compared to none. The more advanced stage at diagnosis was consistently associated with decreased odds of comorbidity incidence for all levels of severity. Moving from most to least deprived areas, the odds of having higher levels of comorbidity decreases. Compared to breast cancer, the odds of having higher levels of comorbidity decreased for brain/CNS (mild, moderate, and severe), soft tissue (mild and moderate) and skin (mild) cancers. Conversely, the odds of having higher levels of comorbidity (compared with none) increases for lung (mild, moderate, and severe), female genital organs, and blood cancers (moderate and severe), and head and neck, urinary tract, and skin (severe) cancers. For other disease sites including digestive organs, male genital organs, and endocrine cancers, the odds were comparable to breast cancer.

**Fig. 6:**
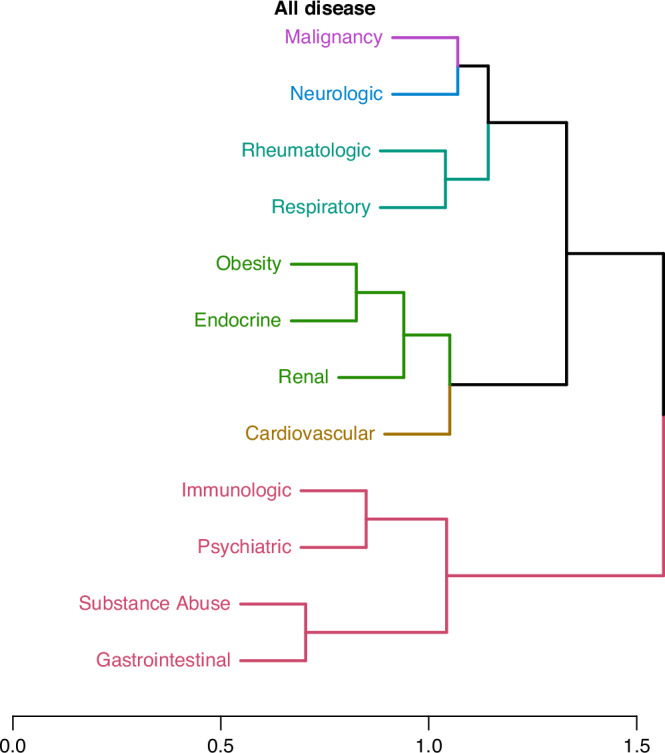
Analysis of clustering including all patients with cancer in this study to identify the presence of multiple medical conditions occurring simultaneously. The arrangement of the 12 organ system conditions into two primary clusters indicates their most frequent co-occurrence.

The hierarchical cluster analysis from the full cohort displayed in Fig. 6 shows the 12 organ system conditions grouped into two main clusters which co-occur most frequently. The upper cluster further grouped into 2 clusters which include malignancy, neurologic, rheumatologic, and respiratory as one cluster and endocrine, cardiovascular, renal, and obesity conditions in the other cluster. The bottom main cluster includes substance abuse, psychiatric, gastrointestinal, and immunologic diseases.

Supplementary Table 7 shows the characteristics of patients with ACE-27 scores analyzed, as well as those without ACE-27 who were excluded from the study. Patients without ACE-27 tend to be younger, exhibit a slightly higher proportion of males, reside in less deprived regions, and are more likely to present with blood, skin, and head and neck cancers. Conversely, they are less likely to have diagnoses of lung and breast cancer.

Among the analyzed data (*n* = 77,149), the majority of the patients were from Greater Manchester (40,048; 51.9%), followed by Cheshire and Merseyside (26,769; 34.7%) and Lancashire and South Cumbria (2490; 3.2%), which together comprised the catchment area for The Christie (data not shown). The remaining patients were either from other regions (2467; 3.2%) or had district postcodes that could not be retrieved for regional categorization (5376; 7.0%). The Supplementary Material Table 8 and Supplementary Fig. 7 present a comparison of gender, age, and socio-economic status of patients from The Christie (*n* = 77,149) analyzed in this study, alongside population data from regions within The Christie catchment area: Cheshire and Merseyside (*n* = 26,109), Greater Manchester (*n* = 23,204), and Lancashire and South Cumbria (*n* = 15,958), as well as aggregate data from England (*n* = 490,959) obtained from NDRS. Notably, the gender distribution at The Christie shows a higher proportion of female patients compared to both its catchment areas and the overall population of England. In terms of age, The Christie has a higher percentage of patients under 70 years old compared to England and all three catchment areas. Conversely, The Christie has a lower proportion of patients aged 80 and above compared to both England and its catchment areas. The deprivation levels at The Christie are similar to those in its catchment area; however, there is a striking difference in the most deprived regions, with The Christie and its catchment areas showing a higher percentage of deprivation compared to England. These findings suggest that while The Christie may reflect the demographics of its immediate catchment area—particularly where deprivation levels are similar—the generalizability of the findings to the broader population of England may be limited.

## Discussion

We investigated the association between comorbidity burden and patients demographic, cancer site, and socio-economic factors in a large cohort of cancer patients treated at a specialist cancer center in the UK. One of the findings of our study was that the incidence and severity of overall ACE-27 scores increase with age across all deprivation quintiles and was highly correlated with ECOG performance status. Moreover, our study revealed a consistent trend of lower severity of comorbidities for patients living in less deprived areas. We observed that the prevalence of moderate and severe comorbidity scores increased when comparing crude to age-standardized rates, while the prevalence of no or mild comorbidity decreased. These findings underscore the influence of socio-economic factors on the burden of comorbidity among cancer patients and highlight the need to tackle wider issues of social and economic inequality as an integral part of any strategy to improve cancer treatment and outcomes.

There is a debate in the literature regarding the impact of comorbidity severity on the diagnosis of cancer, with some arguing that more regular contact with health services for comorbidity management leads to earlier cancer discovery, with the counter-argument being that comorbidities may mask early cancer symptoms, leading to diagnosis at a later stage [3–5]. Our findings indicate a consistent association between more advanced cancer stages and a reduced incidence of specific organ system comorbidities, as well as a higher level of overall comorbidity. This data can be interpreted to support the argument that more contact with health services to manage severe comorbid conditions means there is a greater chance of cancers being discovered at an early stage. This could be also explained by the higher frequency of imaging tests in patients followed up for comorbidities.

The majority of existing studies have limitations in terms of their scope, focusing primarily on specific cancer cohorts, such as colon, rectum, lung, or Hodgkin lymphoma [10], or on a limited range of cancers, including gastrointestinal, breast, lung, sarcoma, urogenital cancer, or other types of underrepresented cancers [11]. In contrast, our study provides a more representative sample of the entire population, which strengthens the generalizability of our findings. Our study uncovered notable disparities in the overall burden of ACE-27 comorbidity across various cancer sites. Specifically, we found that brain/CNS and endocrine gland cancers had the highest percentage of patients without any comorbidity at diagnosis, whereas lung and urinary tract cancers had the lowest percentage. Conversely, lung and skin cancers exhibited the highest percentage of patients with a severe overall comorbidity score, while endocrine glands and breast cancers had the lowest percentage. Age is associated with increased comorbidity incidence and given the different age profiles for the above disease groups, it is difficult to determine whether the association with disease group is entirely independent of age. However, as both factors show association when included in the multivariable analysis, these findings suggest that the differing comorbidity profiles among different cancer types should be considered during patient management and care.

It is noteworthy that previous malignancy and neurologic conditions consistently cluster across all cancer sites (data not shown), except for female and male genital organs. A comparable pattern was evident in the case of substance abuse and gastrointestinal conditions. Moreover, cardiovascular, endocrine, and obesity conditions frequently co-occur across all cancer sites, except for digestive organ cancer (data not shown). These results provide insights into the interconnected relationships among different organ system conditions within the context of cancer.

The Christie NHS Foundation Trust, a leading cancer specialist center, treats approximately 60,000 patients annually, including 12,500 new patients each year [27]. From our collected data from 01/01/2014 – 15/12/2022, The Christie treated a total of 103,262 new cases over this 9-year period, of which 77,149 had ACE-27 scores and 26,099 did not (see Supplementary Material Table 7 and Supplementary Material Fig. 1). This data is consistent with the number of new patients treated at The Christie during the same period.

It is important to acknowledge the limitations of our study. We relied on real-world data, which may have potential biases inherent in its collection. The Christie’s patient population appears more aligned with regional data (The Northwest), indicating that the trends observed within its catchment area could be more representative of the local demographic and health landscape than those of England. Thus, comparisons between The Christie and its catchment areas are likely more valid than comparisons to national data (Supplementary Table 8 and Supplementary Fig. 7). Moreover, differences exist between patients with and without available ACE-27 scores treated at The Christie (Supplementary Table 7), highlighting potential biases in our study population and caution needed in generalizing our findings.

The comorbidity score in our study applies to the period preceding the initiation of cancer treatment at The Christie, within the study period, specifically noting the worst score as it was recorded on the day of a patient’s first consultation prior to treatment commencement. Our results do not capture the dynamic nature of comorbidities. It’s important to interpret our findings within this context, and recognize the potential limitations imposed by the static nature of the comorbidity scores. Moreover, in our study, ACE-27 scores were derived primarily from data provided by referring physicians working in secondary care settings. However, this approach may not fully capture comorbidities managed exclusively in primary care, potentially limiting their representation in our analysis. Such limitations could impact the generalizability of our findings, highlighting the importance of future research incorporating primary care data to achieve a more comprehensive understanding of comorbidity profiles in cancer patients.

The ACE-27 comorbidity score used in our study may have its own limitations, and further research is needed to validate its applicability and reliability in different populations. One notable concern with ACE-27 is its inability to account for all comorbidities, some of which could significantly influence a patient’s performance status or quality of life. For instance, joint conditions may be overlooked by ACE-27, potentially impacting the findings. In our study, the ACE-27 includes a category for ‘previous malignancy’, representing a history of cancer before the episode being considered in this study and does not include the current diagnosis. It should be noted that if a recording of ‘previous malignancy’ is made, it is not possible to determine if the cancer in the study is a recurrence of the historic cancer or new primary cancer. Moreover, reverse causality, where cancer or cancer treatment may influence the development or worsening of comorbidities, is a plausible scenario for patients with previous malignancies. It is possible that either treatment for a previous cancer, or treatment prior to referral to The Christie, may have caused comorbidity. However, the majority of patients referred to The Christie are treatment naïve, which reduces the impact of this scenario on our results. Still, it should be noted that our dataset lacks the granularity to quantify or assess the specific impact of prior cancer treatment on comorbidity development.

In interpreting our findings, it is also important to consider the inherent circularity introduced by The Index of Multiple Deprivation assessment of health. The Index of Multiple Deprivation incorporates health indicators as part of its composite measure, which may alter our observed association between deprivation and comorbidity. This integration suggests that aspects of health are both predictors and outcomes within the index itself. Future research could explore the individual dimensions of deprivation to better disentangle these interrelated factors more effectively. Lastly, this is a retrospective cross-sectional study reporting observed associations and not causal relationships.

Understanding the incidences of comorbidity can guide healthcare providers in planning for the known resource implications of managing cancer patients with comorbidities. For example, in our cohort, cardiovascular comorbidity was the most prevalent condition across all cancer sites, indicating the need for further research on the impact on toxicity and outcomes of cancer patients with pre-existing cardiac conditions. This also underlines the importance of introducing cardio-oncology services to effectively manage and mitigate potential cardiovascular complications in cancer patients. These data, along with studies linking comorbidity incidence to poorer cancer treatment outcomes highlight the importance of considering relative comorbidity burdens when developing effective cancer care plans. Additionally, addressing socio-economic inequalities should be an integral part of healthcare improvement strategies. Further research into the interactions between comorbidity burden and cancer incidence, diagnosis, treatment, and outcomes is warranted.

## Supplementary information

Supplementary material figures

Supplementary material tables

## Data Availability

Data can be made available on request.

## References

[1] Søgaard M, Thomsen RW, Bossen KS, Sørensen HT, Nørgaard M. The Impact of Comorbidity on Cancer Survival: A Review. Clin Epidemiol. 2013;5:3–29. 10.2147/clep.s47150. 24227920 10.2147/CLEP.S47150 PMC3820483

[2] Jakovljević M, Ostojić L. Comorbidity and Multimorbidity in Medicine Today: Challenges and Opportunities for Bringing Separated Branches of Medicine Closer to Each Other. Psychiatr Danubina. 2013;25:18–28. 23806971

[3] Gurney J, Sarfati D, Stanley J. The Impact of Patient Comorbidity on Cancer Stage at Diagnosis. Br J Cancer. 2015;113:1375–80. 10.1038/bjc.2015.355. 26461060 10.1038/bjc.2015.355 PMC4815795

[4] Mounce LTA, Price S, Valderas JM, Hamilton W. Comorbid Conditions Delay Diagnosis of Colorectal Cancer: A Cohort Study Using Electronic Primary Care Records. Br J Cancer. 2017;116:1536–43. 10.1038/bjc.2017.127. 28494470 10.1038/bjc.2017.127 PMC5518856

[5] Terret C, Castel-Kremer E, Albrand G, Droz JP. Effects of Comorbidity on Screening and Early Diagnosis of Cancer in Elderly People. Lancet Oncol. 2009;10:80–7. 10.1016/s1470-2045(08)70336-x. 19111248 10.1016/S1470-2045(08)70336-X

[6] Piccirillo JF, Feinstein AR. Clinical Symptoms and Comorbidity: Significance for the Prognostic Classification of Cancer. Cancer. 1996;77:834–42. 10.1002/(SICI)1097-0142(19960301)775<834::AID-CNCR5>3.0.CO;2-E. 8608472

[7] Extermann M, Overcash J, Lyman GH, Parr J, Balducci L. Comorbidity and Functional Status Are Independent in Older Cancer Patients. J Clin Oncol. 1998;16:1582–7. 10.1200/jco.1998.16.4.1582. 9552069 10.1200/JCO.1998.16.4.1582

[8] Siembida EJ, Smith AW, Potosky AL, Graves KD, Jensen RE. Examination of Individual and Multiple Comorbid Conditions and Health-Related Quality of Life in Older Cancer Survivors. Qual Life Res. 2021;30:1119–29. 10.1007/s11136-020-02713-0. 33447956 10.1007/s11136-020-02713-0 PMC7808400

[9] Gallagher EJ, LeRoith D. Obesity and Diabetes: The Increased Risk of Cancer and Cancer-Related Mortality. Physiological Rev. 2015;95:727–48. 10.1152/physrev.00030.2014. 10.1152/physrev.00030.2014 PMC4491542 26084689

[10] Fowler H, Belot A, Ellis L, Maringe C, Luque-Fernandez MA, Njagi EN, et al. Comorbidity Prevalence among Cancer Patients: A Population-Based Cohort Study of Four Cancers. BMC Cancer. 2020;20:2 10.1186/s12885-019-6472-9. 31987032 10.1186/s12885-019-6472-9 PMC6986047

[11] Balic M, Hilbe W, Gusel S, Fiegl M, Ludwig H, Mayrbäurl B, et al. Prevalence of Comorbidity in Cancer Patients Scheduled for Systemic Anticancer Treatment in Austria. memo Mag Eur Med Oncol. 2019;12:290–6. 10.1007/s12254-019-00542-7.

[12] Tsilidis KK, Kasimis JC, Lopez DS, Ntzani EE, Ioannidis JP. Type 2 Diabetes and Cancer: Umbrella Review of Meta-Analyses of Observational Studies. BMJ. 2015;350:g7607 10.1136/bmj.g7607. 25555821 10.1136/bmj.g7607

[13] Sarfati D, Koczwara B, Jackson C. The Impact of Comorbidity on Cancer and Its Treatment. CA Cancer J Clin. 2016;66:337–50. 10.3322/caac.21342. 26891458 10.3322/caac.21342

[14] Duma N, Kothadia SM, Azam TU, Yadav S, Paludo J, Vera Aguilera J, et al. Characterization of Comorbidities Limiting the Recruitment of Patients in Early Phase Clinical Trials. Oncologist. 2019;24:96–102. 10.1634/theoncologist.2017-0687. 30413668 10.1634/theoncologist.2017-0687 PMC6324635

[15] Klepin HD, Pitcher BN, Ballman KV, Kornblith AB, Hurria A, Winer EP, et al. Comorbidity, Chemotherapy Toxicity, and Outcomes among Older Women Receiving Adjuvant Chemotherapy for Breast Cancer on a Clinical Trial: Calgb 49907 and Calgb 361004 (Alliance). J Oncol Pr. 2014;10:e285–92. 10.1200/jop.2014.001388. 10.1200/JOP.2014.001388 PMC4161730 25074878

[16] Nguyen SM, Pham AT, Nguyen LM, Cai H, Tran TV, Shu XO, et al. Chemotherapy-Induced Toxicities and Their Associations with Clinical and Non-Clinical Factors among Breast Cancer Patients in Vietnam. Curr Oncol. 2022;29:8269–84. 10.3390/curroncol29110653. 36354713 10.3390/curroncol29110653 PMC9689154

[17] Chao C, Page JH, Yang SJ, Rodriguez R, Huynh J, Chia VM. History of Chronic Comorbidity and Risk of Chemotherapy-Induced Febrile Neutropenia in Cancer Patients Not Receiving G-Csf Prophylaxis. Ann Oncol. 2014;25:1821–9. 10.1093/annonc/mdu203. 24915871 10.1093/annonc/mdu203

[18] Piccirillo JF, Tierney RM, Costas I, Grove L, Spitznagel JEL. Prognostic Importance of Comorbidity in a Hospital-Based Cancer Registry. JAMA. 2004;291:2441–7. 10.1001/jama.291.20.2441. 15161894 10.1001/jama.291.20.2441

[19] Sarfati D. Review of Methods Used to Measure Comorbidity in Cancer Populations: No Gold Standard Exists. J Clin Epidemiol. 2012;65:924–33. 10.1016/j.jclinepi.2012.02.017. 22739245 10.1016/j.jclinepi.2012.02.017

[20] Adult Co-Morbidity Evaluation (Ace-27) Uk Values. Available from: https://www.rcplondon.ac.uk/file/3058/download.

[21] Sarfati D, Gurney J, Lim BT, Bagheri N, Simpson A, Koea J, et al. Identifying Important Comorbidity among Cancer Populations Using Administrative Data: Prevalence and Impact on Survival. Asia Pac J Clin Oncol. 2016;12:e47–56. 10.1111/ajco.12130. 24354451 10.1111/ajco.12130

[22] McLean G, Gunn J, Wyke S, Guthrie B, Watt GC, Blane DN, et al. The Influence of Socioeconomic Deprivation on Multimorbidity at Different Ages: A Cross-Sectional Study. Br J Gen Pract. 2014;64:e440–7. 10.3399/bjgp14X680545. 24982497 10.3399/bjgp14X680545 PMC4073730

[23] McLennan D, Noble S, Nobel M, Plunkett E, Wright G, Gutacker N. The English Indices of Deprivation: Technical Report”, Ministry of Housing, Communities and Local Government. 2019. Available from: https://www.gov.uk/government/publications/english-indices-of-deprivation-2019-technical-report.

[24] National Disease Registration Service. 2024. Available from: https://nhsd-ndrs.shinyapps.io/cancers_by_diagnosis_trust/.

[25] Wolfenden HH. On the Methods of Comparing the Moralities of Two or More Communities, and the Standardization of Death-Rates. J R Stat Soc. 1923;86:399–411. 10.2307/2341622.

[26] Everitt B, Landau S, Leese M, Stahl D. Cluster Analysis. Wiley Series in Probability and Statistics, Chichester, West Sussex, UK; 2011. 10.1002/9780470977811.

[27] The Christie Nhs Foundation Trust. 2024. Available from: https://en.wikipedia.org/wiki/The_Christie_NHS_Foundation_Trust.

