# Correction: Comorbidity in patients with cancer treated at The Christie

**DOI:** 10.1038/s41416-024-02884-4

**Published:** 2024-10-21

**Authors:** Azadeh Abravan, Corinne Faivre-Finn, Fabio Gomes, Marcel van Herk, Gareth Price

**Affiliations:** 1 Division of Cancer Sciences, The University of Manchester, Manchester, United Kingdom; 2 The Christie NHS Foundation Trust, Manchester, United Kingdom

**Keywords:** Oncology, Diseases

Correction to: *British Journal of Cancer* 10.1038/s41416-024-02838-w, published online 04 September 2024

In Fig. 5 of this article, the first age range was originally listed as ‘40–40’ rather than ‘40–49’; the figure should have appeared as shown below.
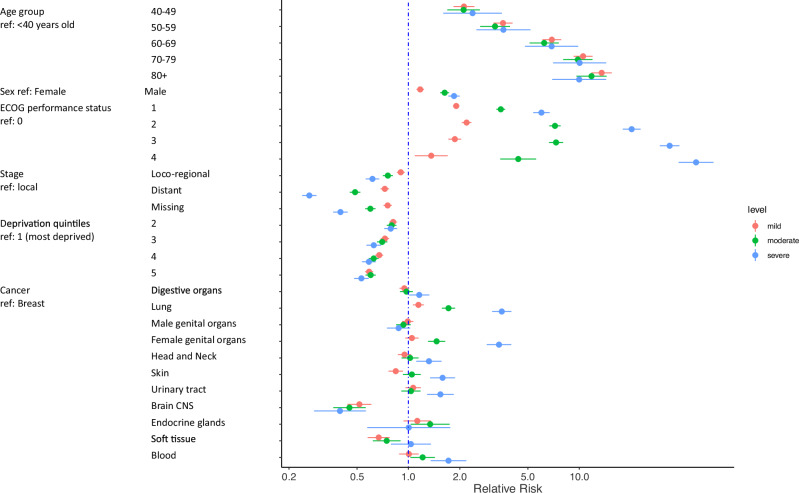


The original article has been updated.

