# Supplementary material for: Comorbidity in patients with cancer treated at The Christie
Source: Br J Cancer. 2024 Sep 4;131(8):1279–89. doi: 10.1038/s41416-024-02838-w (PMC11473959; doi:10.1038/s41416-024-02838-w)

Supplementary Material Figure 1: Flowchart illustrating data selection process. This flowchart depicts the sequential steps taken to refine the dataset for the study. Initially, available data including cancer ICD-10 code, age, gender, and Index of Multiple Deprivation were collected from January 1, 2010, to December 15, 2022. Then, patients with available ACE-27 scores and ECOG performance status essential for this study were selected. Subsequent refinement involved selecting the earliest date for patients with multiple date of ACE-27 assessment, and the highest ACE-27 score and ECOG performance status for patients with multiple recordings for the same date. As shown in Supplementary Material Figure 2, we began consistently collecting structured patient data in 2014. To minimize bias, we excluded patients diagnosed between 01/01/2010 and 01/01/2014.The final cohort, consisting of unique patients meeting all criteria, totaled 77,149 individuals. This rigorous selection process ensured the inclusion of a representative cohort for comprehensive analysis in the study. * To compare patients with ACE-27 analyzed from January 1, 2014, to December 15, 2022, we utilized a unique cohort of patients without ACE-27 during the same time period (n = 26,099). Please refer to Supplementary Material Table 7 for additional details. ICD-10: International Classification of Diseases 10th Revision; ACE-27: Adult Comorbidity Evaluation 27; ECOG performance status: Eastern Cooperative Oncology Group performance status

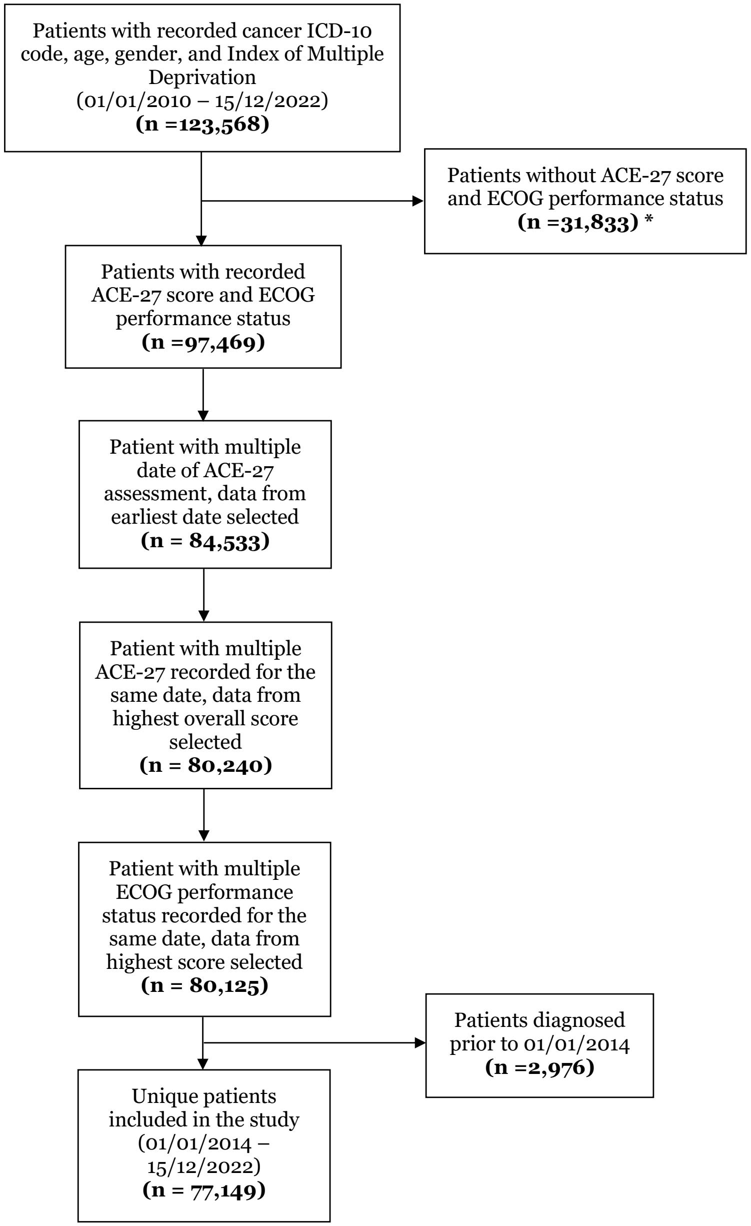

Supplementary Material Figure 2: Distribution of unique patients with cancer available at The Christie healthcare system between 2010-2022 (n=80,125), illustrating the consistent collection of structured data for ACE-27 scores over time, starting from 2014. ACE-27: Adult Comorbidity Evaluation 27

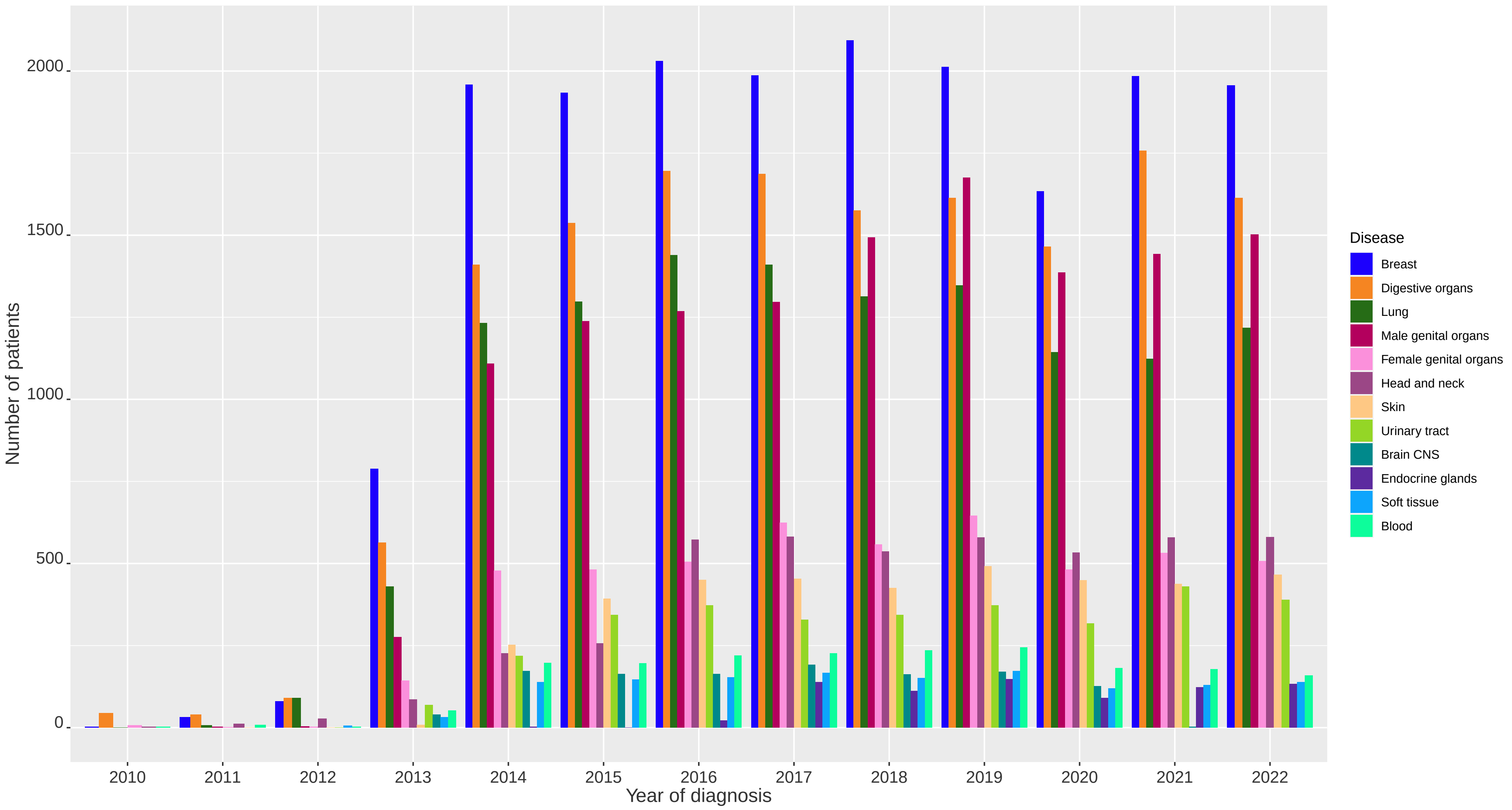

Supplementary Material Figure 3: Distribution of overall ACE-27 comorbidity score across cancer sites. Remarkably, the data reveals substantial discrepancies in the overall burden of ACE-27 comorbidity across various cancer sites. This observation highlights the considerable heterogeneity in comorbidity prevalence and underscores the importance of comprehending these disparities for tailored cancer care strategies. ACE-27: Adult Comorbidity Evaluation 27

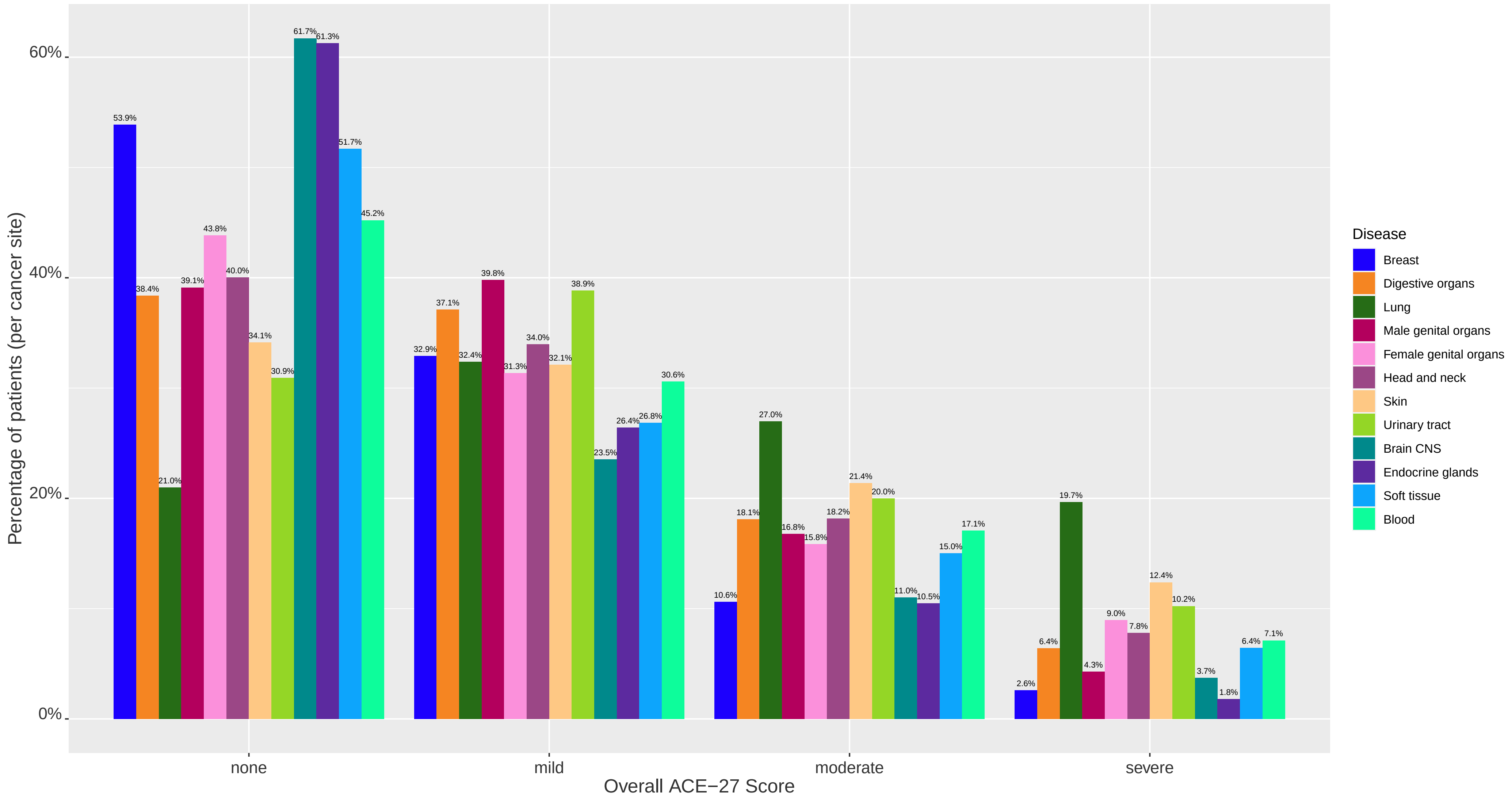

Supplementary Material Figure 4: Age-standardized (left) and crude (right) prevalence of overall ACE-27 score stratified by deprivation quintiles for all patients. Moving from the most to the least deprived regions, a decline in the severity of comorbidities is evident, observed in both age-standardized and crude prevalence rates. Age-standardized data indicated an elevation in the prevalence of moderate and severe comorbidity scores, coupled with a reduction in the prevalence of none or mild comorbidity in each deprivation quintile. This shift is attributed to the higher occurrence of severe comorbidities in older patients, a demographic with relatively fewer individuals. This observation underscores the potential influence of age and socio-economic factors on the distribution of comorbidities within a population.ACE-27: Adult Comorbidity Evaluation 27

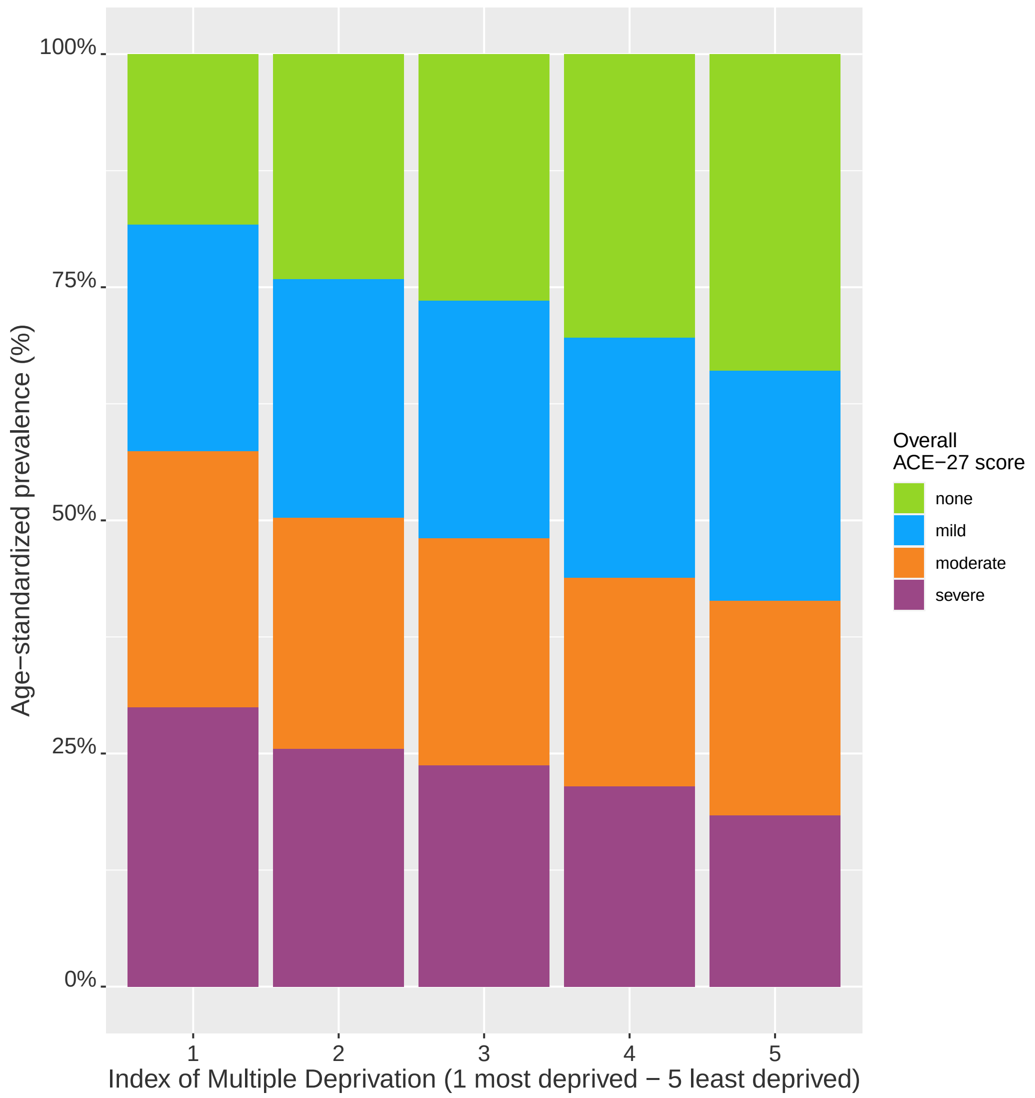

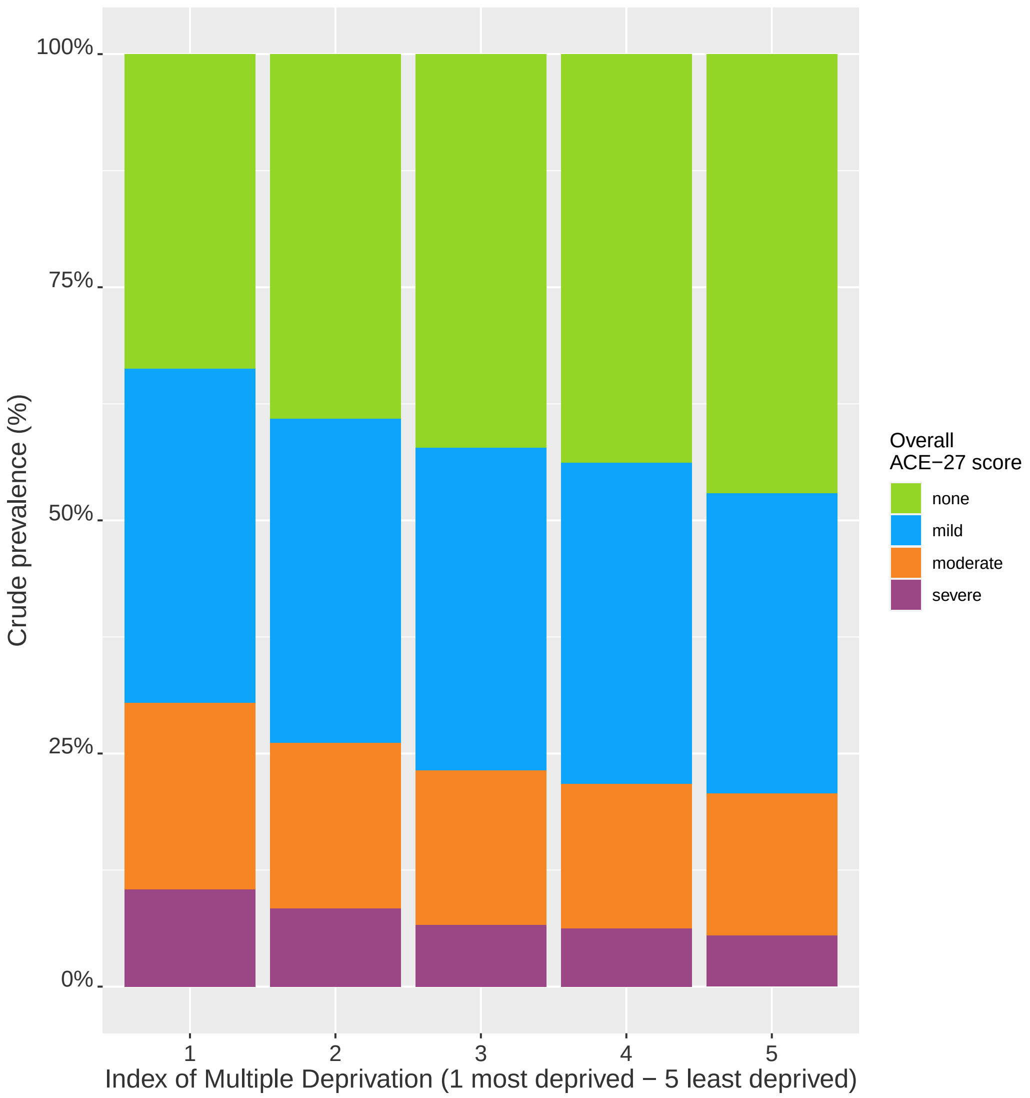

Supplementary Material Figure 5: Age-adjusted prevalence of the 12 conditions per cancer sites in females and males. Notably, cardiovascular comorbidity, including hypertension, emerges as the most widespread condition among all cancer sites in both genders. The prevalence of other conditions varies across different cancer sites and genders, with a higher prevalence observed in males. Note that a patient can have more than one condition.

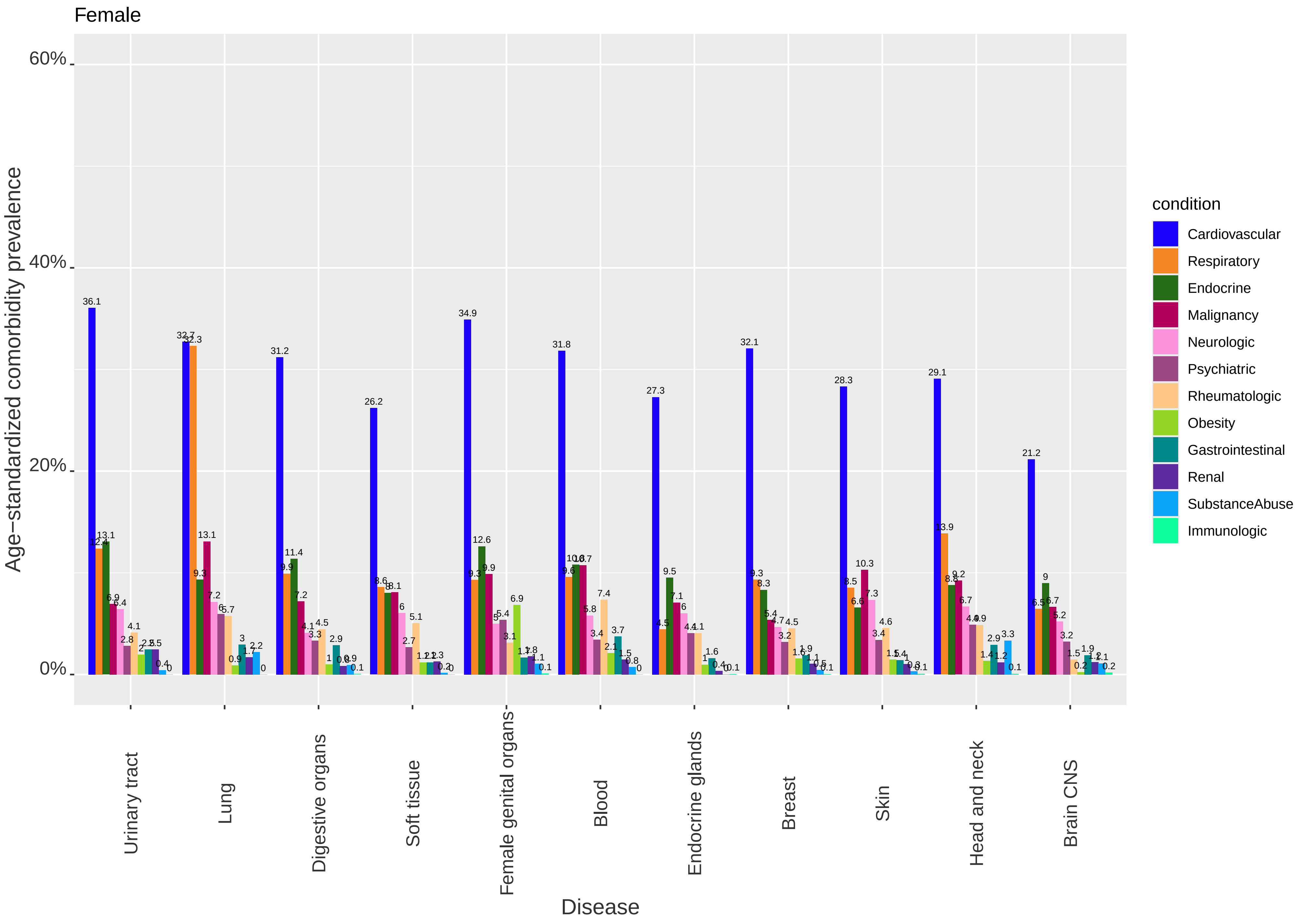

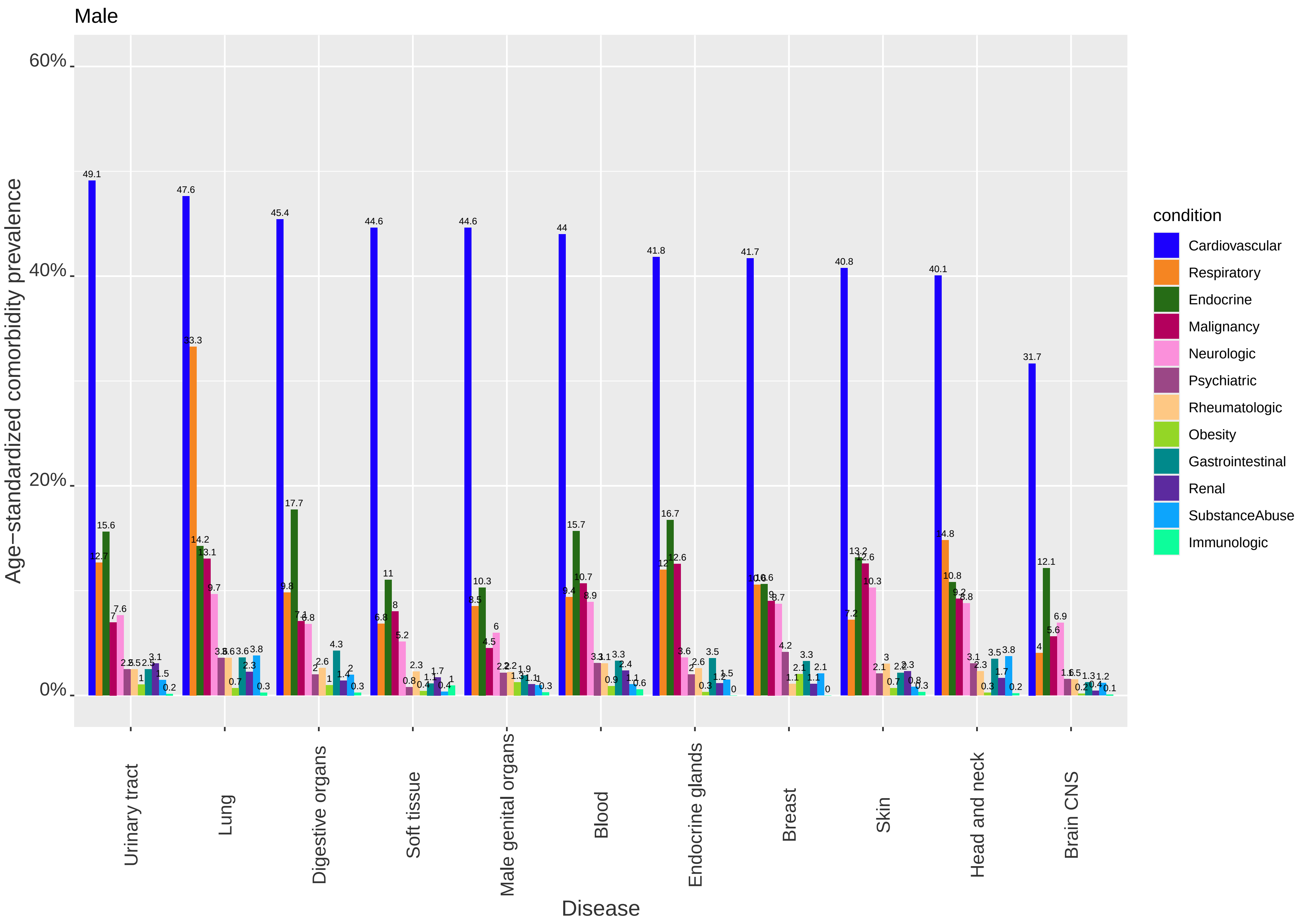
Male
Age−standardized comorbidity prevalence
60%
40%
20%
0%
Disease
Urinary tract
Lung
Digestive organs
Soft tissue
Male genital organs
Blood
Endocrine glands
Breast
Skin
Head and neck
Brain CNS
condition
Cardiovascular
Respiratory
Endocrine
Malignancy
Neurologic
Psychiatric
Rheumatologic
Obesity
Gastrointestinal
Renal
SubstanceAbuse
Immunologic

Supplementary Material Figure 6: Age-adjusted prevalence of 7 cardiovascular conditions per cancer sites in females and males. In general, males had a higher prevalence of these conditions. Hypertension emerged as the predominant cardiac condition across the cancer sites. Note that a patient can have more than one condition.

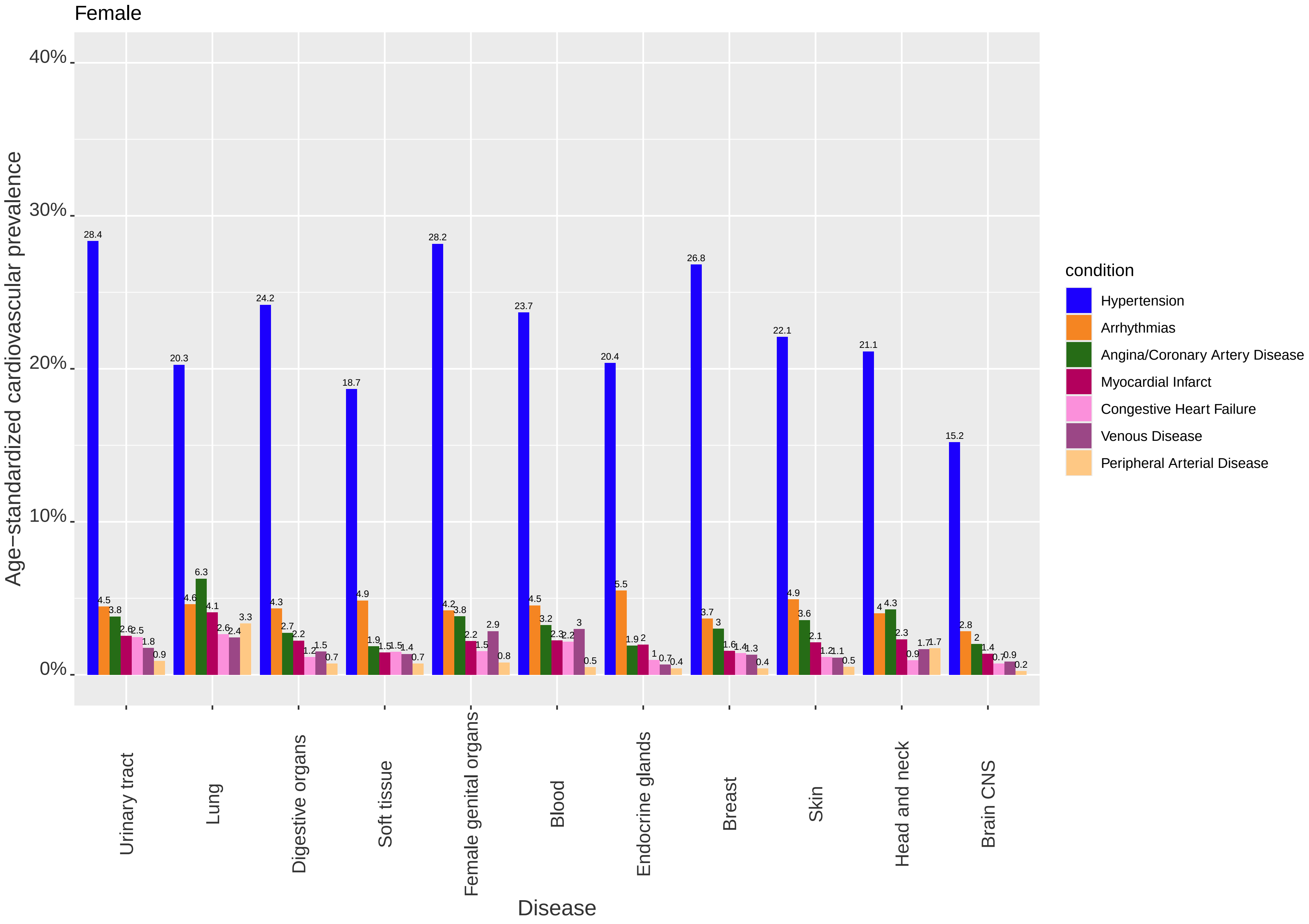

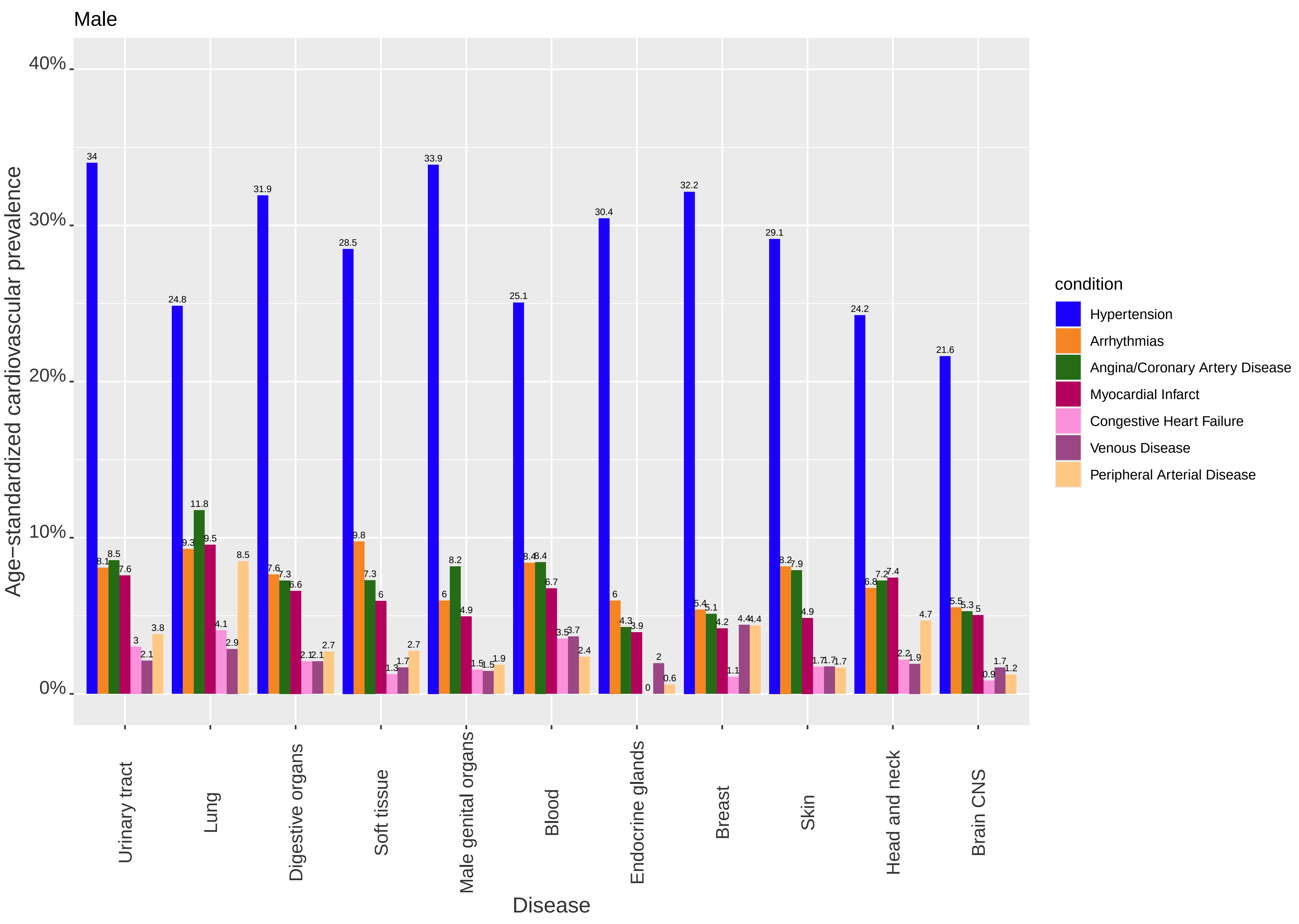
Male
Age−standardized cardiovascular prevalence
40%
30%
20%
10%
0%
34
8.1
8.5
7.6
3
2.1
3.8
24.8
9.3
11.8
9.5
4.1
2.9
8.5
31.9
7.6
7.3
6.6
2.1
2.1
2.7
28.5
9.8
7.3
6
1.3
1.7
2.7
33.9
6
8.2
4.9
1.5
1.5
1.9
25.1
8.4
8.4
6.7
3.5
3.7
2.4
30.4
6
4.3
3.9
0
2
0.6
32.2
5.4
5.1
4.2
1.1
4.4
4.4
29.1
8.2
7.9
4.9
1.7
1.7
1.7
24.2
6.8
7.2
7.4
2.2
1.9
4.7
21.6
5.5
5.3
5
0.9
1.7
1.2
Urinary tract
Lung
Digestive organs
Soft tissue
Male genital organs
Blood
Endocrine glands
Breast
Skin
Head and neck
Brain CNS
Disease
condition
Hypertension
Arrhythmias
Angina/Coronary Artery Disease
Myocardial Infarct
Congestive Heart Failure
Venous Disease
Peripheral Arterial Disease

Supplementary Material Figure 7: Percentage comparison of The Christie cohort analyzed in study with data provided by the National Disease Registration Service (NDRS) for England, as well as the catchment area of The Christie, which includes Greater Manchester, Cheshire and Merseyside, and Lancashire and South Cumbria Care Alliance in 2021. The number of patients per group can be found in Supplementary Material Table 8.

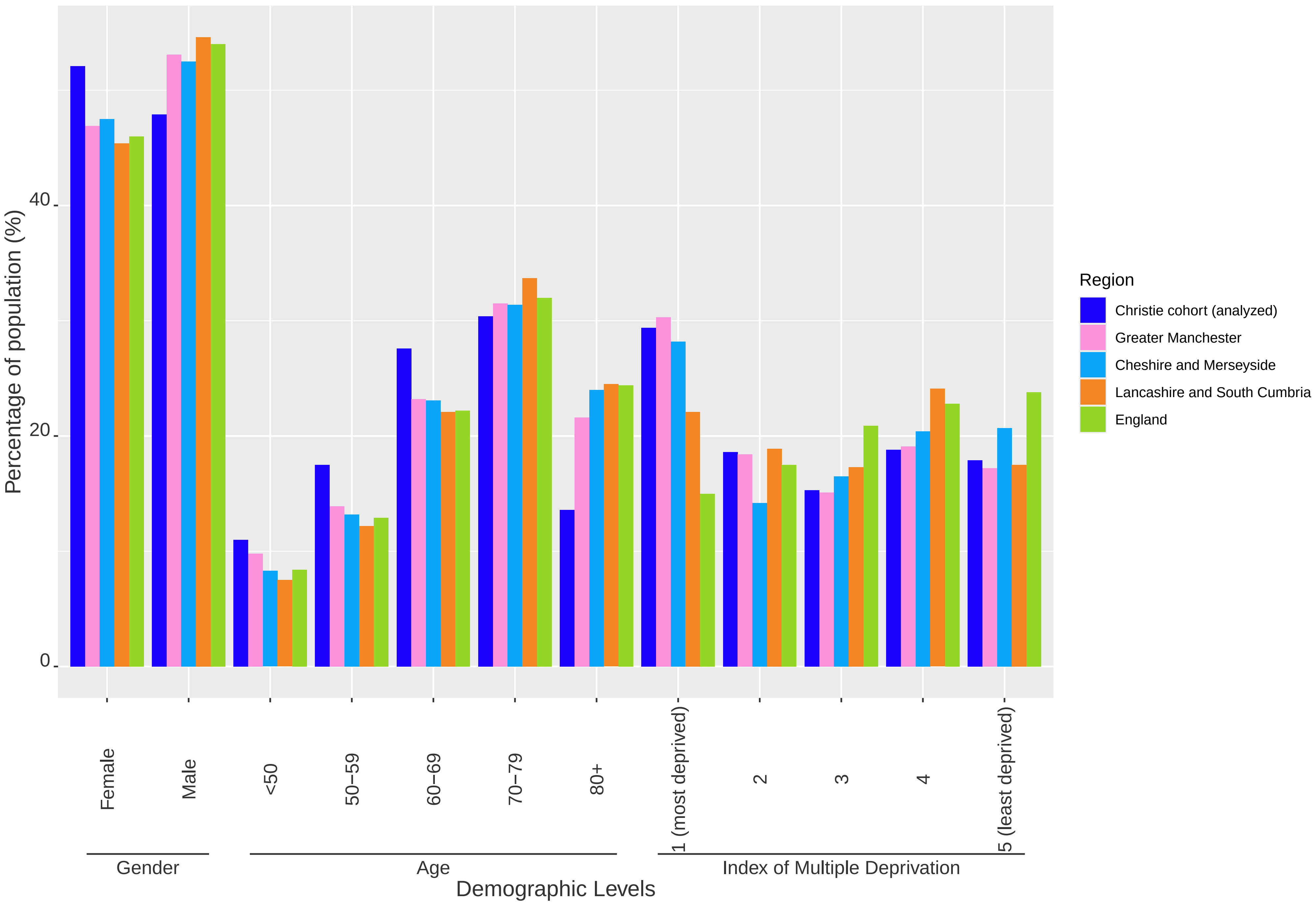

Supplement: Supplementary file 1 — Supplementary material figures [file 41416_2024_2838_MOESM1_ESM.pdf]
