# Supplementary material for: Comorbidity in patients with cancer treated at The Christie
Source: Br J Cancer. 2024 Sep 4;131(8):1279–89. doi: 10.1038/s41416-024-02838-w (PMC11473959; doi:10.1038/s41416-024-02838-w)
Supplement: Supplementary file 2 — Supplementary material tables [file 41416_2024_2838_MOESM2_ESM.docx]

Supplementary Material Table 1: Disease sites were classified based on the International Classification of Diseases; 10th Revision (ICD-10) codes provided by the World Health Organization (WHO)^^[[1]](#footnote-1)^^. The ICD-10 coding system allows for standardized classification and coding of diseases, providing a comprehensive framework for categorizing different disease sites.

| Disease site | WHO ICD-10 codes |
| --- | --- |
| Head and Neck | C0-C14, C30, C32, C76 |
| Digestive organs | C15-C26 |
| Lung | C33, C34, C37, C38 |
| Skin | C43, C44 |
| Soft tissue | C45-C49 |
| Breast | C50 |
| Female genital organs | C51- C58 |
| Male genital organs | C60-C63 |
| Urinary tract | C64-C68 |
| Brain CNS | C71, C72 |
| Endocrine glands | C73-C75 |
| Blood | C81-C86, C88, C90-C96 |

Supplementary Material Table 2-6: Univariable and multivariable logistic regression of ACE-27 organs systems with the incidence of 5% or higher based on the full cohort. ECOG performance status: Eastern Cooperative Oncology Group performance status

| **Cardiovascular system** |  | **Absent** | **Present** | **OR (univariable)** | **OR (multivariable)** |
| --- | --- | --- | --- | --- | --- |
| Age group | <40 | 2938 (97.8) | 67 (2.2) | - | - |
|  | 40-49 | 5051 (92.0) | 437 (8.0) | 3.79 (2.94-4.97, p<0.001) | 3.80 (2.95-4.98, p<0.001) |
|  | 50-59 | 10800 (80.2) | 2661 (19.8) | 10.80 (8.52-13.95, p<0.001) | 9.93 (7.83-12.82, p<0.001) |
|  | 60-69 | 13501 (63.4) | 7794 (36.6) | 25.31 (20.01-32.61, p<0.001) | 21.30 (16.83-27.46, p<0.001) |
|  | 70-79 | 11420 (48.8) | 11994 (51.2) | 46.05 (36.42-59.32, p<0.001) | 35.98 (28.43-46.39, p<0.001) |
|  | 80+ | 4066 (38.8) | 6420 (61.2) | 69.24 (54.65-89.33, p<0.001) | 51.01 (40.19-65.91, p<0.001) |
| Patient gender | Female | 27401 (68.2) | 12802 (31.8) | - | - |
|  | Male | 20375 (55.1) | 16571 (44.9) | 1.74 (1.69-1.79, p<0.001) | 1.46 (1.41-1.51, p<0.001) |
| ECOG performance status | 0 | 24136 (73.6) | 8656 (26.4) | - | - |
|  | 1 | 14415 (56.1) | 11276 (43.9) | 2.18 (2.11-2.26, p<0.001) | 1.54 (1.48-1.60, p<0.001) |
|  | 2 | 5606 (48.4) | 5980 (51.6) | 2.97 (2.85-3.11, p<0.001) | 1.76 (1.67-1.85, p<0.001) |
|  | 3 | 3181 (49.9) | 3191 (50.1) | 2.80 (2.65-2.96, p<0.001) | 1.57 (1.48-1.67, p<0.001) |
|  | 4 | 438 (61.9) | 270 (38.1) | 1.72 (1.47-2.00, p<0.001) | 1.04 (0.88-1.22, p=0.630) |
| Stage | local | 16500 (59.5) | 11235 (40.5) | - | - |
|  | distant | 8011 (59.5) | 5448 (40.5) | 1.00 (0.96-1.04, p=0.954) | 0.78 (0.75-0.82, p<0.001) |
|  | loco-regional | 10111 (63.9) | 5706 (36.1) | 0.83 (0.80-0.86, p<0.001) | 0.87 (0.83-0.91, p<0.001) |
|  | missing | 13154 (65.3) | 6984 (34.7) | 0.78 (0.75-0.81, p<0.001) | 0.82 (0.78-0.85, p<0.001) |
| Deprivation index quintiles | 1 (most deprived) | 13561 (59.8) | 9115 (40.2) | - | - |
|  | 2 | 8810 (61.3) | 5557 (38.7) | 0.94 (0.90-0.98, p=0.004) | 0.92 (0.88-0.97, p=0.001) |
|  | 3 | 7321 (62.1) | 4469 (37.9) | 0.91 (0.87-0.95, p<0.001) | 0.87 (0.83-0.92, p<0.001) |
|  | 4 | 9116 (62.7) | 5421 (37.3) | 0.88 (0.85-0.92, p<0.001) | 0.82 (0.78-0.86, p<0.001) |
|  | 5 (least deprived) | 8968 (65.1) | 4811 (34.9) | 0.80 (0.76-0.83, p<0.001) | 0.73 (0.70-0.77, p<0.001) |

| **Respiratory system** |  | **Absent** | **Present** | **OR (univariable)** | **OR (multivariable)** |
| --- | --- | --- | --- | --- | --- |
| Age group | <40 | 2929 (97.5) | 76 (2.5) | - | - |
|  | 40-49 | 5179 (94.4) | 309 (5.6) | 2.30 (1.79-2.99, p<0.001) | 2.01 (1.56-2.62, p<0.001) |
|  | 50-59 | 12354 (91.8) | 1107 (8.2) | 3.45 (2.75-4.41, p<0.001) | 2.63 (2.09-3.37, p<0.001) |
|  | 60-69 | 18301 (85.9) | 2994 (14.1) | 6.30 (5.04-8.01, p<0.001) | 4.17 (3.33-5.32, p<0.001) |
|  | 70-79 | 19298 (82.4) | 4116 (17.6) | 8.22 (6.58-10.43, p<0.001) | 4.55 (3.63-5.80, p<0.001) |
|  | 80+ | 8727 (83.2) | 1759 (16.8) | 7.77 (6.20-9.89, p<0.001) | 3.11 (2.47-3.98, p<0.001) |
| Patient gender | Female | 34853 (86.7) | 5350 (13.3) | - | - |
|  | Male | 31935 (86.4) | 5011 (13.6) | 1.02 (0.98-1.07, p=0.298) | 0.86 (0.83-0.90, p<0.001) |
| ECOG performance status | 0 | 30940 (94.4) | 1852 (5.6) | - | - |
|  | 1 | 22024 (85.7) | 3667 (14.3) | 2.78 (2.62-2.95, p<0.001) | 2.67 (2.51-2.84, p<0.001) |
|  | 2 | 8575 (74.0) | 3011 (26.0) | 5.87 (5.51-6.25, p<0.001) | 5.53 (5.16-5.93, p<0.001) |
|  | 3 | 4657 (73.1) | 1715 (26.9) | 6.15 (5.72-6.62, p<0.001) | 6.11 (5.63-6.62, p<0.001) |
|  | 4 | 592 (83.6) | 116 (16.4) | 3.27 (2.66-4.00, p<0.001) | 3.66 (2.95-4.50, p<0.001) |
| Stage | local | 23646 (85.3) | 4089 (14.7) | - | - |
|  | distant | 11508 (85.5) | 1951 (14.5) | 0.98 (0.92-1.04, p=0.506) | 0.56 (0.53-0.60, p<0.001) |
|  | loco-regional | 13518 (85.5) | 2299 (14.5) | 0.98 (0.93-1.04, p=0.555) | 0.83 (0.78-0.88, p<0.001) |
|  | missing | 18116 (90.0) | 2022 (10.0) | 0.65 (0.61-0.68, p<0.001) | 0.49 (0.46-0.52, p<0.001) |
| Deprivation index quintiles | 1 (most deprived) | 18239 (80.4) | 4437 (19.6) | - | - |
|  | 2 | 12267 (85.4) | 2100 (14.6) | 0.70 (0.66-0.74, p<0.001) | 0.76 (0.71-0.80, p<0.001) |
|  | 3 | 10489 (89.0) | 1301 (11.0) | 0.51 (0.48-0.54, p<0.001) | 0.58 (0.54-0.62, p<0.001) |
|  | 4 | 13106 (90.2) | 1431 (9.8) | 0.45 (0.42-0.48, p<0.001) | 0.52 (0.49-0.55, p<0.001) |
|  | 5 (least deprived) | 12687 (92.1) | 1092 (7.9) | 0.35 (0.33-0.38, p<0.001) | 0.43 (0.40-0.46, p<0.001) |

| **Endocrine system** |  | **Absent** | **Present** | **OR (univariable)** | **OR (multivariable)** |
| --- | --- | --- | --- | --- | --- |
| Age group | <40 | 2970 (98.8) | 35 (1.2) | - | - |
|  | 40-49 | 5288 (96.4) | 200 (3.6) | 3.21 (2.27-4.68, p<0.001) | 3.11 (2.19-4.54, p<0.001) |
|  | 50-59 | 12460 (92.6) | 1001 (7.4) | 6.82 (4.94-9.75, p<0.001) | 5.96 (4.31-8.53, p<0.001) |
|  | 60-69 | 18650 (87.6) | 2645 (12.4) | 12.03 (8.75-17.16, p<0.001) | 9.37 (6.80-13.37, p<0.001) |
|  | 70-79 | 19993 (85.4) | 3421 (14.6) | 14.52 (10.56-20.69, p<0.001) | 10.06 (7.30-14.36, p<0.001) |
|  | 80+ | 9020 (86.0) | 1466 (14.0) | 13.79 (10.00-19.70, p<0.001) | 8.35 (6.04-11.96, p<0.001) |
| Patient gender | Female | 36383 (90.5) | 3820 (9.5) | - | - |
|  | Male | 31998 (86.6) | 4948 (13.4) | 1.47 (1.41-1.54, p<0.001) | 1.31 (1.25-1.37, p<0.001) |
| ECOG performance status | 0 | 30786 (93.9) | 2006 (6.1) | - | - |
|  | 1 | 22223 (86.5) | 3468 (13.5) | 2.39 (2.26-2.54, p<0.001) | 1.96 (1.85-2.09, p<0.001) |
|  | 2 | 9545 (82.4) | 2041 (17.6) | 3.28 (3.07-3.50, p<0.001) | 2.52 (2.34-2.70, p<0.001) |
|  | 3 | 5211 (81.8) | 1161 (18.2) | 3.42 (3.16-3.70, p<0.001) | 2.62 (2.41-2.85, p<0.001) |
|  | 4 | 616 (87.0) | 92 (13.0) | 2.29 (1.82-2.85, p<0.001) | 1.90 (1.51-2.38, p<0.001) |
| Stage | local | 24843 (89.6) | 2892 (10.4) | - | - |
|  | distant | 11575 (86.0) | 1884 (14.0) | 1.40 (1.31-1.49, p<0.001) | 1.02 (0.95-1.09, p=0.610) |
|  | loco-regional | 13974 (88.3) | 1843 (11.7) | 1.13 (1.06-1.21, p<0.001) | 1.08 (1.01-1.15, p=0.017) |
|  | missing | 17989 (89.3) | 2149 (10.7) | 1.03 (0.97-1.09, p=0.390) | 0.97 (0.91-1.03, p=0.325) |
| Deprivation index quintiles | 1 (most deprived) | 19360 (85.4) | 3316 (14.6) | - | - |
|  | 2 | 12641 (88.0) | 1726 (12.0) | 0.80 (0.75-0.85, p<0.001) | 0.83 (0.78-0.89, p<0.001) |
|  | 3 | 10590 (89.8) | 1200 (10.2) | 0.66 (0.62-0.71, p<0.001) | 0.71 (0.66-0.76, p<0.001) |
|  | 4 | 13162 (90.5) | 1375 (9.5) | 0.61 (0.57-0.65, p<0.001) | 0.66 (0.61-0.70, p<0.001) |
|  | 5 (least deprived) | 12628 (91.6) | 1151 (8.4) | 0.53 (0.50-0.57, p<0.001) | 0.59 (0.55-0.64, p<0.001) |

| **Previous malignancy** |  | **Absent** | **Present** | **OR (univariable)** | **OR (multivariable)** |
| --- | --- | --- | --- | --- | --- |
| Age group | <40 | 2947 (98.1) | 58 (1.9) | - | - |
|  | 40-49 | 5331 (97.1) | 157 (2.9) | 1.50 (1.11-2.04, p=0.009) | 1.45 (1.08-1.99, p=0.016) |
|  | 50-59 | 12907 (95.9) | 554 (4.1) | 2.18 (1.67-2.90, p<0.001) | 2.04 (1.56-2.71, p<0.001) |
|  | 60-69 | 19825 (93.1) | 1470 (6.9) | 3.77 (2.92-4.97, p<0.001) | 3.33 (2.58-4.39, p<0.001) |
|  | 70-79 | 21014 (89.7) | 2400 (10.3) | 5.80 (4.50-7.63, p<0.001) | 4.71 (3.65-6.21, p<0.001) |
|  | 80+ | 9030 (86.1) | 1456 (13.9) | 8.19 (6.34-10.80, p<0.001) | 5.82 (4.49-7.71, p<0.001) |
| Patient gender | Female | 37010 (92.1) | 3193 (7.9) | - | - |
|  | Male | 34044 (92.1) | 2902 (7.9) | 0.99 (0.94-1.04, p=0.653) | 0.88 (0.83-0.92, p<0.001) |
| ECOG performance status | 0 | 31254 (95.3) | 1538 (4.7) | - | - |
|  | 1 | 23279 (90.6) | 2412 (9.4) | 2.11 (1.97-2.25, p<0.001) | 1.69 (1.58-1.82, p<0.001) |
|  | 2 | 10237 (88.4) | 1349 (11.6) | 2.68 (2.48-2.89, p<0.001) | 1.95 (1.79-2.12, p<0.001) |
|  | 3 | 5637 (88.5) | 735 (11.5) | 2.65 (2.41-2.90, p<0.001) | 1.86 (1.68-2.06, p<0.001) |
|  | 4 | 647 (91.4) | 61 (8.6) | 1.92 (1.45-2.48, p<0.001) | 1.36 (1.03-1.77, p=0.026) |
| Stage | local | 25532 (92.1) | 2203 (7.9) | - | - |
|  | distant | 12391 (92.1) | 1068 (7.9) | 1.00 (0.93-1.08, p=0.978) | 0.83 (0.76-0.89, p<0.001) |
|  | loco-regional | 14748 (93.2) | 1069 (6.8) | 0.84 (0.78-0.91, p<0.001) | 0.83 (0.76-0.89, p<0.001) |
|  | missing | 18383 (91.3) | 1755 (8.7) | 1.11 (1.04-1.18, p=0.002) | 1.05 (0.98-1.12, p=0.175) |
| Deprivation index quintiles | 1 (most deprived) | 20889 (92.1) | 1787 (7.9) | - | - |
|  | 2 | 13239 (92.1) | 1128 (7.9) | 1.00 (0.92-1.08, p=0.919) | 1.01 (0.93-1.09, p=0.863) |
|  | 3 | 10912 (92.6) | 878 (7.4) | 0.94 (0.86-1.02, p=0.153) | 0.95 (0.87-1.04, p=0.269) |
|  | 4 | 13380 (92.0) | 1157 (8.0) | 1.01 (0.94-1.09, p=0.785) | 1.02 (0.94-1.10, p=0.702) |
|  | 5 (least deprived) | 12634 (91.7) | 1145 (8.3) | 1.06 (0.98-1.14, p=0.144) | 1.09 (1.00-1.18, p=0.044) |

| **Neurologic system** |  | **Absent** | **Present** | **OR (univariable)** | **OR (multivariable)** |
| --- | --- | --- | --- | --- | --- |
| Age group | <40 | 2983 (99.3) | 22 (0.7) | - | - |
|  | 40-49 | 5389 (98.2) | 99 (1.8) | 2.49 (1.60-4.06, p<0.001) | 2.26 (1.44-3.69, p=0.001) |
|  | 50-59 | 13057 (97.0) | 404 (3.0) | 4.20 (2.80-6.65, p<0.001) | 3.08 (2.05-4.89, p<0.001) |
|  | 60-69 | 20193 (94.8) | 1102 (5.2) | 7.40 (4.97-11.65, p<0.001) | 4.30 (2.88-6.79, p<0.001) |
|  | 70-79 | 21457 (91.6) | 1957 (8.4) | 12.37 (8.33-19.45, p<0.001) | 5.56 (3.73-8.77, p<0.001) |
|  | 80+ | 9077 (86.6) | 1409 (13.4) | 21.05 (14.16-33.13, p<0.001) | 6.37 (4.26-10.08, p<0.001) |
| Patient gender | Female | 38026 (94.6) | 2177 (5.4) | - | - |
|  | Male | 34130 (92.4) | 2816 (7.6) | 1.44 (1.36-1.53, p<0.001) | 1.30 (1.22-1.38, p<0.001) |
| ECOG performance status | 0 | 32176 (98.1) | 616 (1.9) | - | - |
|  | 1 | 24140 (94.0) | 1551 (6.0) | 3.36 (3.05-3.69, p<0.001) | 3.07 (2.78-3.39, p<0.001) |
|  | 2 | 10129 (87.4) | 1457 (12.6) | 7.51 (6.82-8.28, p<0.001) | 6.52 (5.88-7.24, p<0.001) |
|  | 3 | 5213 (81.8) | 1159 (18.2) | 11.61 (10.49-12.87, p<0.001) | 10.50 (9.39-11.75, p<0.001) |
|  | 4 | 498 (70.3) | 210 (29.7) | 22.03 (18.38-26.34, p<0.001) | 22.45 (18.56-27.10, p<0.001) |
| Stage | local | 25752 (92.9) | 1983 (7.1) | - | - |
|  | distant | 12593 (93.6) | 866 (6.4) | 0.89 (0.82-0.97, p=0.007) | 0.49 (0.45-0.54, p<0.001) |
|  | loco-regional | 14830 (93.8) | 987 (6.2) | 0.86 (0.80-0.94, p<0.001) | 0.78 (0.72-0.85, p<0.001) |
|  | missing | 18981 (94.3) | 1157 (5.7) | 0.79 (0.73-0.85, p<0.001) | 0.61 (0.56-0.66, p<0.001) |
| Deprivation index quintiles | 1 (most deprived) | 20988 (92.6) | 1688 (7.4) | - | - |
|  | 2 | 13442 (93.6) | 925 (6.4) | 0.86 (0.79-0.93, p<0.001) | 0.94 (0.86-1.02, p=0.152) |
|  | 3 | 11056 (93.8) | 734 (6.2) | 0.83 (0.75-0.90, p<0.001) | 0.98 (0.89-1.07, p=0.624) |
|  | 4 | 13664 (94.0) | 873 (6.0) | 0.79 (0.73-0.86, p<0.001) | 0.96 (0.88-1.05, p=0.340) |
|  | 5 (least deprived) | 13006 (94.4) | 773 (5.6) | 0.74 (0.68-0.81, p<0.001) | 0.94 (0.86-1.03, p=0.195) |

Supplementary Material Table 7: Characteristics of unique patients with and without ACE-27 scores (01/01/2014-15/12/2022). * Analyzed is this study.

| label | levels | With ACE-27 scores*  (n=77,149) | Without ACE-27 scores  (n=26,099) |
| --- | --- | --- | --- |
| Gender | Female | 40203 (52.1) | 12084 (46.3) |
|  | Male | 36946 (47.9) | 14015 (53.7) |
| Age (year) | Mean (SD) | 65.9 (13.1) | 62.6 (17.1) |
| Age group | <40 | 3005 (3.9) | 2718 (10.4) |
|  | 40-49 | 5488 (7.1) | 2163 (8.3) |
|  | 50-59 | 13461 (17.4) | 4423 (16.9) |
|  | 60-69 | 21295 (27.6) | 6520 (25.0) |
|  | 70-79 | 23414 (30.3) | 6794 (26.0) |
|  | 80+ | 10486 (13.6) | 3481 (13.3) |
| Disease | Blood | 1840 (2.4) | 1695 (6.5) |
|  | Bone | - | 94 (0.4) |
|  | Brain CNS | 1151 (1.5) | 506 (1.9) |
|  | Breast | 17591 (22.8) | 2102 (8.1) |
|  | Digestive organs | 14352 (18.6) | 5414 (20.7) |
|  | Endocrine glands | 772 (1.0) | 581 (2.2) |
|  | Female genital organs | 4813 (6.2) | 1543 (5.9) |
|  | Head and Neck | 4446 (5.8) | 2770 (10.6) |
|  | Lung | 11524 (14.9) | 1755 (6.7) |
|  | Male genital organs | 12409 (16.1) | 3591 (13.8) |
|  | Skin | 3816 (4.9) | 4367 (16.7) |
|  | Soft tissue | 1318 (1.7) | 704 (2.7) |
|  | Urinary tract | 3117 (4.0) | 977 (3.7) |
| Deprivation index quintiles | 1 (most deprived) | 22676 (29.4) | 6357 (25.2) |
|  | 2 | 14367 (18.6) | 4431 (17.6) |
|  | 3 | 11790 (15.3) | 4066 (16.1) |
|  | 4 | 14537 (18.8) | 5040 (20.0) |
|  | 5 (least deprived) | 13779 (17.9) | 5351 (21.2) |

Supplementary Material Table 8: Demographics of patients from The Christie analyzed in the study, along with broader population data from regions within The Christie catchment area: Greater Manchester, Cheshire and Merseyside, and Lancashire and South Cumbria Care Alliance, as well as England. *Data were obtained from the National Disease Registration Service (NDRS) for all cancer cases diagnosed in 2021^[[2]](#footnote-2)^.

| label | levels | Christie patients with ACE-27 (n=77,149) (analyzed) | England  (n=490959) * | Cheshire and Merseyside (n=26109) * | Greater Manchester (n=23204) * | Lancashire and South Cumbria (n= 15958) * |
| --- | --- | --- | --- | --- | --- | --- |
| Gender | Female | 40203 (52.1) | 225997 (46) | 12412 (47.5) | 10893 (46.9) | 7250 (45.4) |
|  | Male | 36946 (47.9) | 264962 (54) | 13697 (52.5) | 12311 (53.1) | 8707 (54.6) |
| Age group | <50 | 8494 (11) | 41433 (8.4) | 2162 (8.3) | 2267 (9.8) | 1201 (7.5) |
|  | 50-59 | 13464 (17.4) | 63391 (12.9) | 3451 (13.2) | 3233 (13.9) | 1943 (12.2) |
|  | 60-69 | 21297 (27.6) | 109236 (22.2) | 6035 (23.1) | 5394 (23.2) | 3527 (22.1) |
|  | 70-79 | 23419 (30.4) | 156969 (32) | 8206 (31.4) | 7302 (31.5) | 5372 (33.7) |
|  | 80+ | 10489 (13.6) | 119930 (24.4) | 6255 (24) | 5008 (21.6) | 3915 (24.5) |
| Deprivation index quintiles | 1 (most deprived) | 22680 (29.4) | 73581 (15) | 7363 (28.2) | 7026 (30.3) | 3533 (22.1) |
|  | 2 | 14371 (18.6) | 85851 (17.5) | 3699 (14.2) | 4270 (18.4) | 3020 (18.9) |
|  | 3 | 11793 (15.3) | 102806 (20.9) | 4315 (16.5) | 3501 (15.1) | 2761 (17.3) |
|  | 4 | 14539 (18.8) | 112000 (22.8) | 5324 (20.4) | 4427 (19.1) | 3846 (24.1) |
|  | 5 (least deprived) | 13780 (17.9) | 116721 (23.8) | 5408 (20.7) | 3980 (17.2) | 2798 (17.5) |

1. <https://icd.who.int/browse10/2010/en#/> [↑](#footnote-ref-1)
2. <https://nhsd-ndrs.shinyapps.io/cancers_by_diagnosis_trust/> [↑](#footnote-ref-2)
